# Geometry and cooling effects on an induction self-heating insert for rapid mold temperature control: Experimental, Multiphysics, and artificial neural network-based analysis

**DOI:** 10.1371/journal.pone.0359849

**Published:** 2026-10-27

**Authors:** Thanh Trung Do, Tran Minh The Uyen, Pham Son Minh

**Affiliations:** Faculty of Mechanical Engineering, HCM City University of Technology and Engineering, Ho Chi Minh City, Vietnam; King Mongkut's University of Technology North Bangkok, THAILAND

## Abstract

Control of mold-cavity surface temperature strongly affects filling, premature frozen-layer formation, and cycle time in thin-wall injection molding. This study evaluates an induction self-heating insert with a thin heating zone directly coupled to a cooling layer using experiments, a sequentially coupled electromagnetic-thermal model, and an Artificial Neural Network (ANN). The design space contains 25 Taguchi L25 geometries defined by T, W, and L; X and Y coordinates are added so that the ANN predicts the surface temperature field. Three cooling states are considered: No Water (NW), Stagnant Water (SW), and Flowing Water (FW). Experimental ANN targets are the mean of five repeated tests. The simulation reproduced the location of the hot zone; among four cases with reported quantitative differences, three differed by no more than about 10 °C and one showed a peak difference of about 20 °C. To prevent data leakage, all 846 points from each geometry were kept together during geometry-wise validation. The resulting R² values were 0.746, 0.653, and 0.493 for NW, SW, and FW, with Root Mean Square Error (RMSE) values of 17.03, 12.97, and 12.62 °C, respectively. The effects of T, W, and L depended on the cooling state and were not consistently monotonic. A proof-of-concept molding test showed complete filling of a Chip 2 part using Low-Density Polyethylene (LDPE); the mean spacing was 28.6 µm for a nominal 30 µm, and the mean height was 106.6 µm for a nominal 100 µm. These results show that combining insert geometry, cooling-water state, and a surrogate model can support local thermal design, delay frozen-layer formation, and improve filling in injection molding.

## 1. Introduction

In injection molding, mold-cavity surface temperature controls the rate of frozen-layer formation at the polymer-mold interface. When the mold is too cold, the polymer near the wall freezes early, reduces the effective flow area, increases pressure loss, and raises the risk of short shots in thin sections or regions far from the gate. These effects are especially important in micro-injection molding and high-aspect-ratio features [1–3]. Variotherm strategies, including rapid heat cycle molding (RHCM), address this problem by heating the cavity surface rapidly before filling and cooling it afterward instead of keeping the entire mold at a high temperature [4,5]. Local thermal control can therefore improve flow and surface replication while limiting the increase in cycle time.

Induction heating is well suited to this purpose because energy is transferred through a time-varying electromagnetic field and converted into Joule heat in the conductive part. Early studies established simulation and experimental verification of induction-heated mold plates [6,7]. Rapid induction heating has also been used to improve microfeature replication [8,9] and weld-line quality [10,11]. Other dynamic heating methods, such as steam and hot gas, can also raise the mold-surface temperature quickly [12,13], while infrared heating has been applied to micro-injection molding [14,15]. These methods share the same goal: deliver heat to the cavity surface shortly before the hot polymer reaches the mold.

Recent work on thin-wall products has further shown the importance of mold-temperature control. An external induction-heating system combined with insert rotation provided a 5–8 s heating window and improved the performance of thin-wall parts [16]. A local gas-heating system coupled with an Artificial Neural Network and Genetic Algorithm (GA) showed that higher mold temperature can improve the weld-line strength of polyamide 6 (PA6) [17]. Taguchi L25 designs combined with multi-response analysis have also been used to study several injection-molding variables with a controlled number of experiments [18]. However, most previous studies focused on operating conditions, product quality, or the heat-source configuration. The combined effects of insert geometry, cooling state, and coordinate-based temperature-field prediction have rarely been treated within one framework.

Artificial Neural Networks are useful surrogate models for nonlinear multivariable relations, but their reliability depends strongly on the data structure and the train-test split. In thermal problems, ANNs have been used for mixed convection and greenhouse air-temperature prediction [19,20]. Data-driven models have also been applied to motor cooling and spray-water cooling [21,22]. Other studies used neural networks for radio refractivity and discrete-parameter calibration [23,24], while finite-element and state-identification studies highlighted the need for independent validation when the data have internal structure [25, 26]. In material-property prediction and optimization, R² is commonly reported together with error metrics and sensitivity analysis [27–29]. Applications in thermo-mechanical, polymer, and manufacturing problems further show that ANN models can learn multivariable relations [30–32]. Related modeling work also stresses that data-driven predictions should remain linked to the actual process conditions [33–35]. For a temperature field sampled over one insert, a random split of X-Y points can place points from the same geometry in both training and testing sets. A high correlation in that case may not represent the ability to predict a new geometry.

Another gap concerns the cooling stage. Many induction-heating studies mainly address coil distance, coil shape, heating-zone arrangement, current distribution, and temperature uniformity during heating [6,36,37]. Three-dimensional models and electromagnetic material studies also focus mainly on formation and optimization of the hot zone [38–40]. Infrared and hot-gas studies usually emphasize heating rate and filling during injection [14,41,42]. Some RHCM studies include both heating and cooling [5,43,44], but the cooling-water state is rarely treated as an independent design variable together with the geometry of the heating zone. In contrast, cooling-layer studies show that placing coolant in a large-area layer close to the cavity surface can improve heat removal and temperature uniformity [45,46]. One conformal cooling-layer design reduced cooling time by more than 26% compared with conventional drilled channels in a case study [47].

The main contribution of this study is the combined design of the heating and cooling structures. First, the thin insert region is integrated into the induction-current path, so the high-frequency induced current closes through the region that must be heated and Joule heat is generated directly in that functional region [48]. In external-coil systems, electromagnetic coupling depends strongly on the coil-to-part distance and coil geometry [36,38]. The present architecture reduces intermediate energy-transfer steps and therefore provides a physical basis for efficient local heating. Because overall electrical-to-thermal efficiency was not measured directly, this advantage is discussed as a structural feature rather than as a claim of the highest absolute efficiency. Second, many external heating methods use an electromagnetic gap, gas or infrared flow, or a thin insert to reduce thermal mass, but energy must still cross a gap or intermediate layer before reaching the cavity surface [14,16,41]. Here, the heating region is thin and directly adjacent to the cooling-water layer, which shortens the heat-transfer path during both heating and cooling. Third, NW, SW, and FW are studied as separate boundary conditions to quantify the competition between induction heat generation and water cooling. Previous cooling-layer studies support the potential benefits of near-cavity cooling for heat removal and temperature uniformity [45,47], but this study uses that concept as a supporting design feature rather than as a separate cooling-channel benchmark.

Based on this background, the study has four connected objectives. It first examines how the geometric parameters T, W, and L affect the hot zone within a Taguchi L25 design space. It then treats NW, SW, and FW as separate boundary conditions to determine how cooling changes the geometric response. Using the measured temperature fields, a five-input ANN with T, W, L, X, and Y is developed and evaluated by geometry-wise validation, in which all points from an unseen geometry are excluded from training. Finally, the practical meaning of the thermal results is examined through a molding demonstration focused on filling and replication of small features. This framework shifts the design target from simply maximizing temperature toward the combined design of the heating zone, cooling state, and reliable prediction for a new geometry.

## 2. Materials and methods

### 2.1. Experimental design

The study followed an experiment-simulation-data workflow. First, 25 insert geometries were defined by a Taguchi L25 orthogonal array using T, W, and L at five levels. For each geometry, the surface temperature after 5 s of heating was measured with an infrared camera. Each condition was repeated five times, and the mean of the five repetitions was used for analysis. In parallel, a sequentially coupled electromagnetic-thermal model was used to reproduce the temperature distribution. Spatial temperature data were represented by T, W, L, X, and Y to train separate ANN models for NW, SW, and FW. After geometry-wise validation was completed, models retrained with all 25 geometries were used only for response analysis and design maps. Finally, an additional injection-molding test was used as a proof of concept to illustrate filling when the insert was induction-heated before injection.

#### 2.1.1. Induction-heating principle.

The physical principle is shown in Fig 1. A high-frequency alternating source creates a time-varying electromagnetic field. When the conductive insert is placed in this field, the induced electromotive force produces eddy currents within the insert. In the self-heating structure, the high-frequency induced current closes through the thin region that must be heated, so Joule heat is generated in the functional region rather than transferred through an intermediate heater. The basic electromagnetic and thermal relations are:

**Fig 1:**
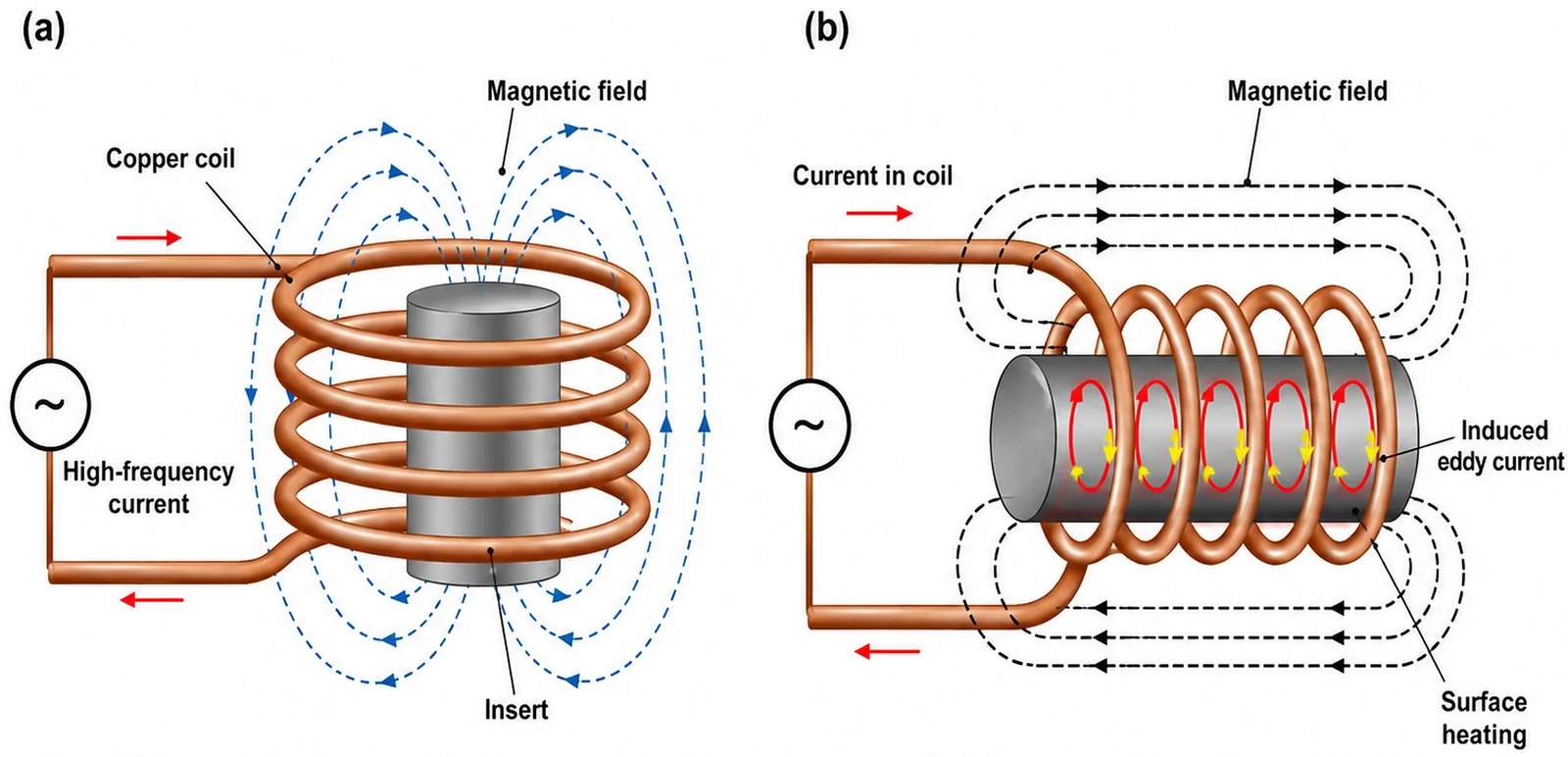
Working principle of induction heating and eddy-current generation in the insert: (a) external high-frequency coil and alternating magnetic field around the insert; (b) induced eddy-current path and localized surface heating in the conductive insert.


$$\begin{array}{c} \nabla \;\times \text{ E }=\;-\partial \text{B}/\partial \text{t} \end{array}$$ (1)

$$\begin{array}{c} \text{J }=\;\sigma \text{E} \end{array}$$ (2)

$$\begin{array}{c} \text{q}\_\text{J }=\text{ J }\cdot \text{ E }=\;{\text{J}}^{\text{2}}/\sigma \end{array}$$ (3)

$$\begin{array}{c} \rho {\text{C}}_{\text{p}}\;\partial \text{T}/\partial \text{t }=\;\nabla \;\cdot \;(\text{k}\nabla \text{T}+\text{ q}\_\text{J} \end{array}$$ (4)

$$\begin{array}{c} \text{q}\_\text{conv }=\text{ h}(\text{T}\_\text{s }-\text{ T}\_\text{f}) \end{array}$$ (5)

where E is the electric-field intensity, B is the magnetic flux density, J is the current density, σ is the electrical conductivity, q_J is the Joule heat-source density, ρ is the density, Cₚ is the specific heat capacity, k is the thermal conductivity, h is the convective heat-transfer coefficient, T_s is the surface temperature, and T_f is the fluid temperature. Equation (4) describes the balance between induction heat generation and heat diffusion in the insert, while Eq (5) represents heat removal by the surrounding medium or the water layer. Because of the skin effect, current density tends to concentrate near the surface and around geometric regions that change the current path. Thus, T, W, and L can affect both the heat-source distribution and the heat-transfer path [6,48].

#### 2.1.2. Insert geometry and Taguchi L25 design.

The experimental insert has overall dimensions of approximately 120 × 90 × 4 mm. The central heating region is parameterized by T, W, and L (Fig 2). T denotes the characteristic thickness of the self-heating region, W its width, and L its length. To describe a temperature field rather than only a peak value, the spatial coordinates X and Y define the measurement positions on the surface. Three X-lines (X1–X3) and three Y-lines (Y1–Y3) are used to compare temperature profiles and construct the ANN dataset.

**Fig 2:**
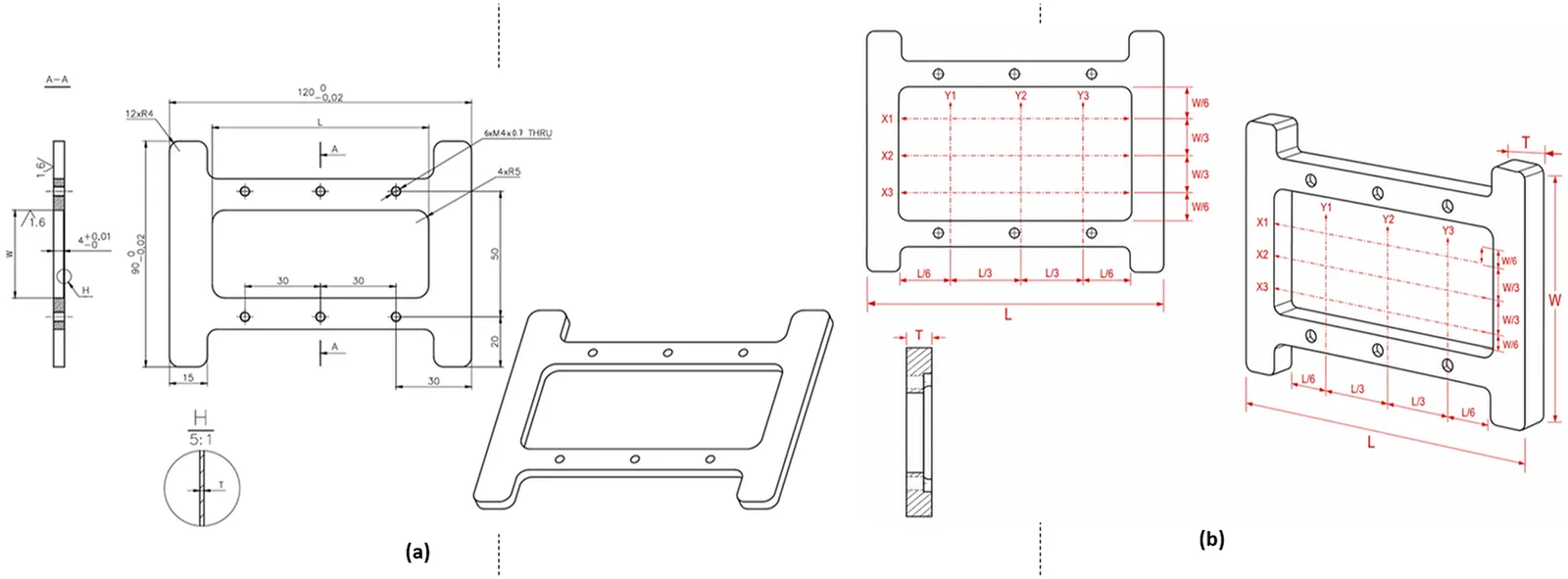
Geometry, key dimensions, and temperature-measurement locations of the self-heating insert: (a) dimensional drawing and 3D view of the insert; (b) definition of L, W, T, and the temperature-measurement lines X1–X3 and Y1–Y3 in 2D and 3D views.

The Taguchi L25 design was selected to study three factors at five levels without testing all 5³ = 125 combinations. The investigated ranges were T = 0.3–1.1 mm, W = 30–40 mm, and L = 76–96 mm (Table 1). The orthogonal array provides a structured geometry space and 25 Geometry IDs for grouped validation. It is not used to infer a causal model outside the investigated range. The complete Taguchi L25 geometry matrix is provided in S1 Table in S1 Data.

**Table 1:** Geometric factors and levels used in the Taguchi L25 design.

Factor	Symbol	Unit	Level 1	Level 2	Level 3	Level 4	Level 5
Heating-zone thickness	T	mm	0.3	0.5	0.7	0.9	1.1
Heating-zone width	W	mm	30	32.5	35	37.5	40
Heating-zone length	L	mm	76	81	86	91	96

Note. T, W, and L denote heating-zone thickness, width, and length, respectively.

### 2.2. Materials, operating parameters, and experimental system

The experimental insert was made of C45 steel. The high-frequency heating unit has a maximum power of 80 kW, a 380 V supply, and a maximum current of 150 A. The operating current reported for this study was 150 A at 20 kHz, and the main heating time used for temperature-field comparison was 5 s (Table 2). The archived COMSOL files were used to recover the numerical formulation, mesh, solver, material-property set, and cooling-boundary settings. The numerical steel domain was represented by a constant soft-iron surrogate property set rather than temperature-dependent C45 properties. The available numerical results are therefore used for thermal-field interpretation and representative experiment-model comparison rather than as a fully recalibrated material model.

**Table 2:** Experimental operating conditions and numerical-model material and thermal-boundary parameters.

Parameter	Value	Unit	Application	Note
Insert material	C45		Experiment	Experimental insert material; numerical steel domain represented separately by a soft-iron surrogate property set
Density	7800	kg/m³	Numerical model	Constant soft-iron surrogate property used in the archived numerical model
Thermal conductivity	20	W/(m·K)	Numerical model	Constant soft-iron surrogate property used in the archived numerical model
Heat capacity	420	J/(kg·K)	Numerical model	Constant soft-iron surrogate property used in the archived numerical model
Electrical conductivity	1.0 × 10⁷	S/m	Numerical model	Constant soft-iron surrogate property used in the archived numerical model
Relative permeability	100	–	Numerical model	Constant soft-iron surrogate property used in the archived numerical model
Induction current	150	A	Study operating condition	Operating current reported for this study
Frequency	20	kHz	Numerical model	Frequency-domain electromagnetic solution
Heating duration	5	s	Experiment/ numerical comparison	Temperature fields compared at t = 5 s
Transient output interval	0.5	s	Simulation	Stored thermal output sequence: 0:0.5:5 s; adaptive internal BDF stepping
NW cooling condition	No water		Cooling condition	Cooling-layer convection boundary disabled
SW heat-transfer coefficient	200	W/(m²·K)	Cooling condition	Prescribed stagnant-water convection boundary; external-fluid temperature 25 °C
FW heat-transfer coefficient	2000	W/(m²·K)	Cooling condition	Prescribed effective forced-convection boundary; external-fluid temperature 25 °C
Numerical steel representation	Soft iron surrogate	–	Numerical model	Constant property set used to represent the C45 insert domain
Initial/ external-fluid temperature	25	°C	Numerical model	298.15 K; used as initial temperature and as the external-fluid temperature for SW/FW convection boundaries

(*) Note. The experimental insert was C45 steel. The numerical steel domain used the constant soft-iron surrogate property set listed above. The cooling-water states were represented by prescribed thermal boundary conditions rather than a resolved CFD model.

Surface temperature was recorded with a FLUKE infrared (IR) camera and processed in SmartView (Fig 3). Additional views of the SmartView interface and the infrared camera are provided in S1 Fig. The camera has a wavelength range of 3–5.4 µm, a scanning interval of 0.1 s, an accuracy of about ±2 °C, and a measurement range of 20–500 °C according to the instrument specification. After the insert was mounted, it was heated for 5 s, and the thermal image was stored and sampled using the defined coordinates. Each geometry/condition was repeated five times. The ANN target field is the mean of the five repetitions. Because the five individual temperature fields are no longer available in the analysis dataset, pointwise standard deviation (SD) and coefficient of variation cannot be reconstructed. The equipment and repeatability information are summarized in Table 3.

**Table 3:** Experimental equipment, thermal measurement conditions, and repeatability.

Category	Parameter	Value	Unit/ format	Note
Induction-heating unit	Control system	Programmable Logic Controller (PLC)		
Induction-heating unit	Maximum power	80	kW	Machine specification
Induction-heating unit	Power supply	380	V	Machine specification
Induction-heating unit	Maximum current	150	A	Machine specification
Induction-heating unit	Cooling	Water		Machine specification
Induction-heating unit	Sensor type	K-Type		Machine specification
Infrared camera	Sensor wavelength	3–5.4	µm	Infrared-camera specification
Infrared camera	Scanning interval	0.1	s	Infrared-camera specification
Infrared camera	Measurement accuracy	±2	°C	Infrared-camera specification
Infrared camera	Temperature range	20–500	°C	Infrared-camera specification
Thermal analysis	Software	SmartView Fluke		Temperature-field recording and extraction
Experimental repeatability	Repetitions per geometry/condition	5	runs	ANN target field is the mean of five repetitions
Experimental repeatability	Individual repeat data	Not available in the retained analysis dataset		Pointwise SD/CV cannot be reconstructed without individual repeat fields

(*) Note. The ANN target temperature field represents the mean of five experimental repetitions for each geometry/condition.

**Fig 3:**
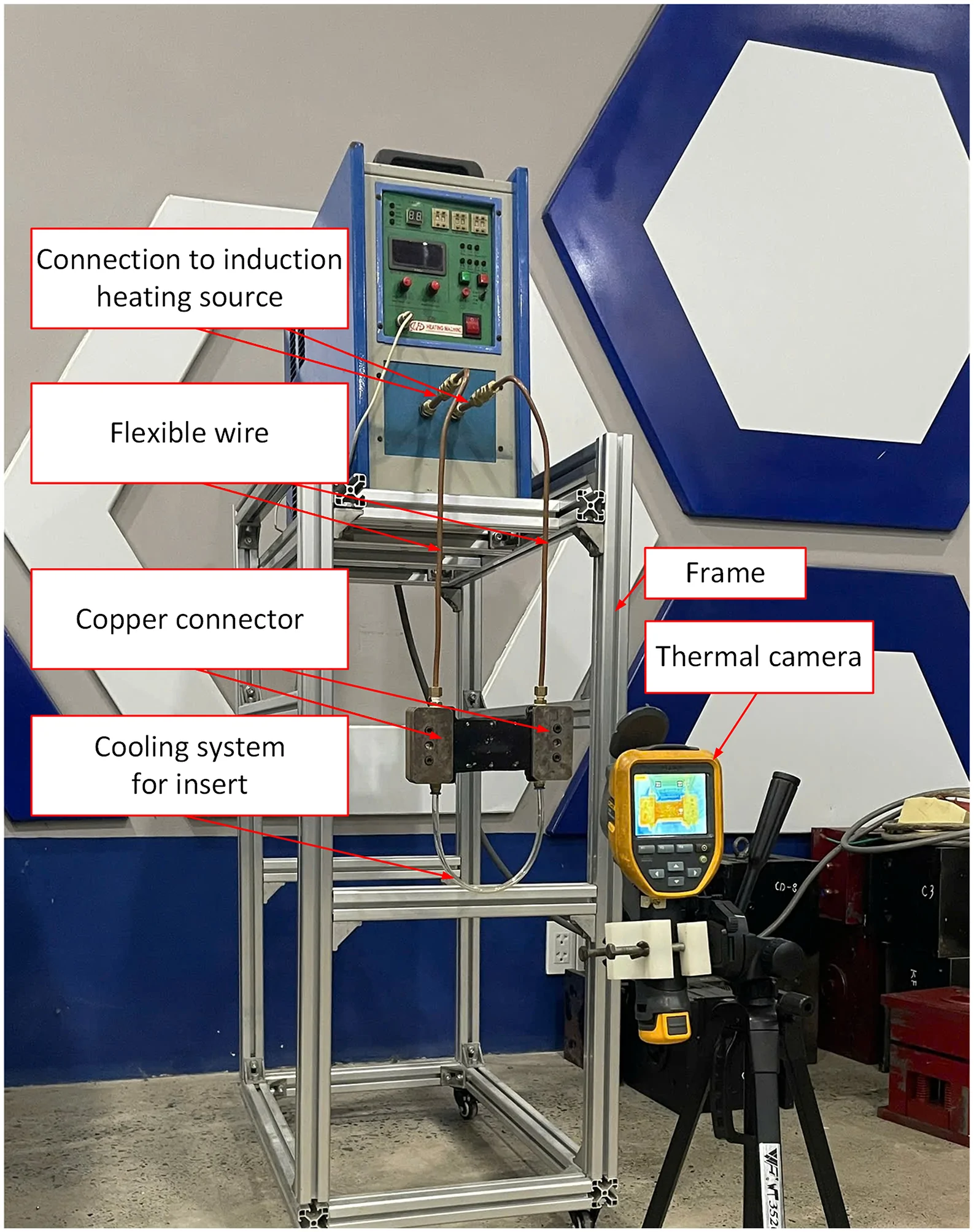
Experimental induction-heating and infrared temperature-measurement system, showing the high-frequency power supply, copper connections, insert fixture, infrared camera, and data-acquisition computer.

### 2.3. Insert assembly and clamping configuration

The insert is clamped between conductive/mechanical blocks and copper components to provide the electrical path and mechanical stiffness during testing (Fig 4). The thin heating region is located next to a water cooling layer, which gives a short heat-transfer path from the hot region to the coolant. Structurally, the same low-thermal-mass region can therefore be heated rapidly before injection and cooled directly after filling. This study uses that feature to examine the effect of water state; it does not claim a quantified cycle-time improvement over a conventional cooling-channel design that was not tested as a control.

**Fig 4:**
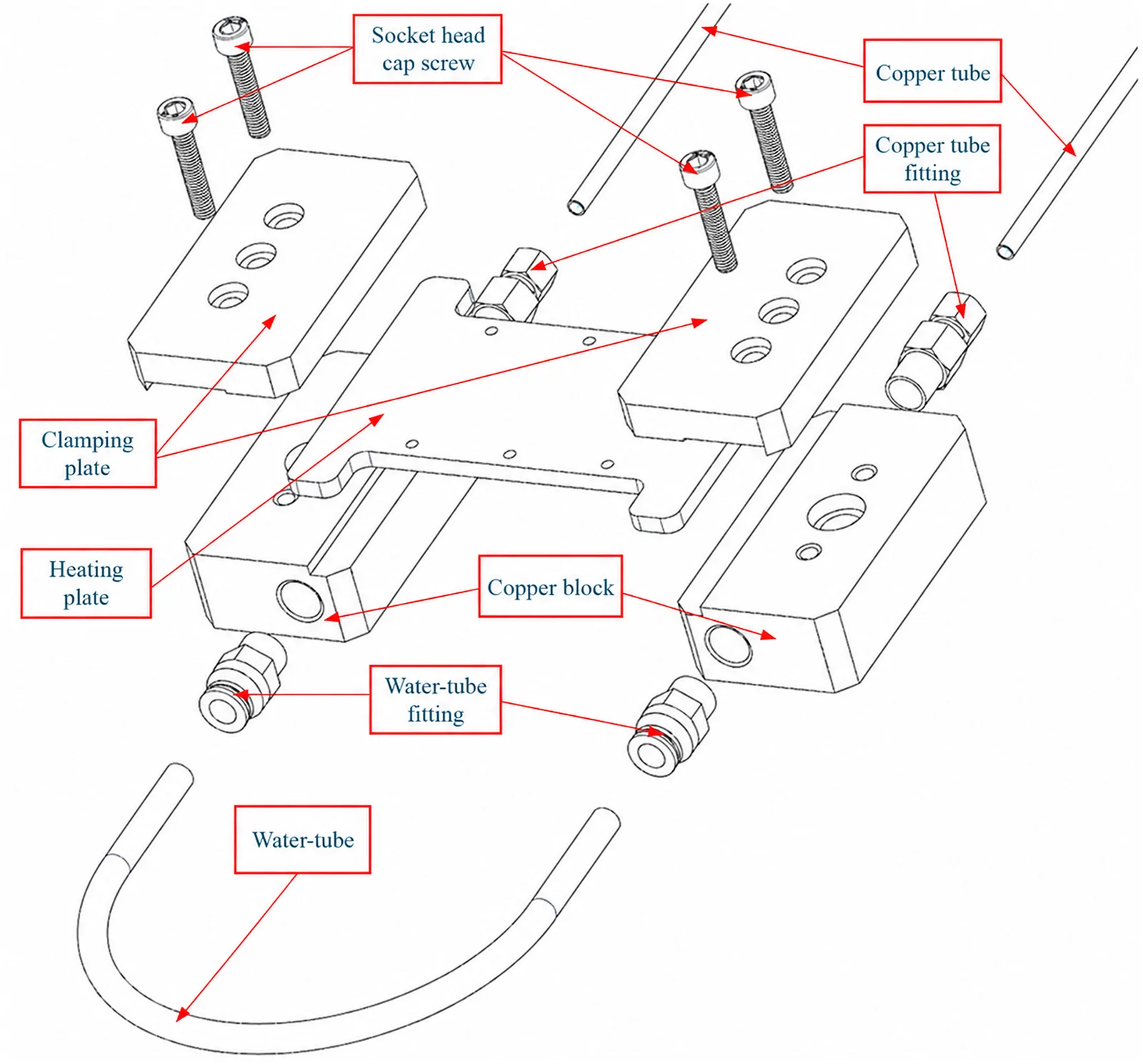
Exploded assembly of the self-heating insert, showing the socket head cap screws, clamping plates, heating plate, copper blocks, copper tubes and fittings, water-tube fittings, and water tube.

### 2.4. Numerical model and boundary conditions

The numerical analysis was implemented in COMSOL Multiphysics 6.3 using Magnetic Fields and Heat Transfer in Solids. The model was sequentially coupled rather than fully two-way coupled. A frequency-domain electromagnetic solution at 20 kHz was first used to determine the electromagnetic loss distribution. The resulting volumetric electromagnetic loss, represented in COMSOL by mf.Qh, was then transferred as the heat-source term to the transient heat-transfer calculation. The electromagnetic and thermal relations are consistent with Eqs (1)-(5), where Joule/electromagnetic loss provides the internal heat source and convection provides the cooling-layer heat loss.

The experimental insert was manufactured from C45 steel, whereas the steel domain in the archived numerical model was represented by a soft-iron surrogate property set. The constant properties used for this domain were density 7800 kg/m³, heat capacity 420 J/(kg·K), thermal conductivity 20 W/(m·K), electrical conductivity 1.0 × 10⁷ S/m, and relative permeability 100. Copper components were also represented with constant properties. Temperature dependence of the electrical and thermal properties was not included in the archived model. Solid-solid interfaces were treated with perfect thermal continuity, and no explicit thermal contact resistance was included. Exterior surfaces without a specified cooling boundary were treated as thermally insulated during the short 5-s heating interval.

A free-tetrahedral mesh was used. The retained mesh settings were a maximum element size of 11.6 mm, a minimum element size of 0.84 mm, a maximum element growth rate of 1.4, a curvature factor of 0.4, and a narrow-region resolution of 0.7. The representative transient thermal model contained approximately 94,356 thermal degrees of freedom plus 43,492 internal degrees of freedom, while the electromagnetic solution contained more than 1.1 million degrees of freedom. The mesh was refined automatically in geometrically sensitive regions, including corners, section transitions, and the thin heating zone (Fig 5).

**Fig 5:**
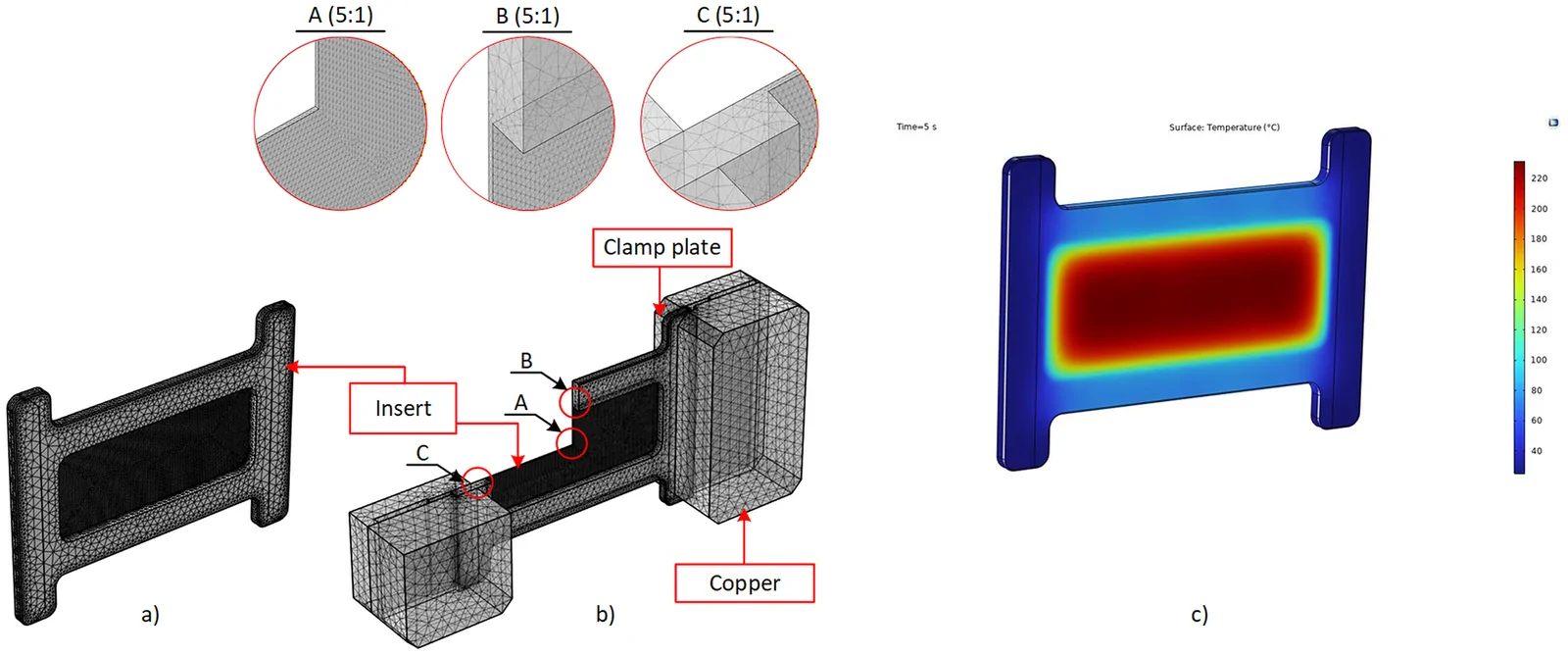
Finite-element model, mesh details, and representative simulated thermal field of the insert: (a) finite-element mesh of the insert; (b) assembled insert-clamp-copper model with enlarged local mesh regions A-C; (c) representative surface-temperature field at t = 5 s.

The transient thermal solution covered 0–5 s and stored output at 0.5-s intervals. Time integration used an adaptive backward differentiation formula (BDF) scheme with an initial time step of approximately 0.001 s and a maximum BDF order of 2. The retained solver settings included a relative tolerance of about 0.01, an absolute tolerance of 1 × 10^−3^, a fully coupled nonlinear solution, a maximum of five nonlinear iterations, and a damping factor of 0.9. A formal mesh-independence study and a separate time-step sensitivity study were not retained with the archived model; therefore, no such independence claim is made in this work.

The numerical model was used to examine hot-zone formation and the effects of geometry and cooling, not to replace measurement. Validation was based on temperature fields and profiles at t = 5 s for representative geometries by comparing simulation with infrared measurements. Because the complete nodal data are no longer available for all cases, full-field numerical MAE and RMSE were not recalculated. Only differences that can be traced directly to the retained results are reported.

### 2.5. Cooling conditions

The three cooling states were retained as NW, SW, and FW, but in the COMSOL model they were represented as prescribed thermal boundary states rather than as a resolved computational-fluid-dynamics flow domain. For NW, the cooling-layer convective boundary was disabled. For SW, the cooling layer was represented by a prescribed convection coefficient h = 200 W/(m²·K) with an external-fluid temperature of 298.15 K (25 °C). For FW, an effective forced-convection boundary was prescribed with h = 2000 W/(m²·K) and the same external-fluid temperature of 298.15 K. Thus, FW in the numerical model denotes the thermal effect of flowing water through an effective forced-convection boundary rather than a directly resolved water-flow field. Flow rate, Reynolds number, and Nusselt number are therefore not model inputs in the present formulation. Separating these three boundary states allows the competition between induction heat generation and heat removal to be evaluated without claiming a hydraulic CFD validation.

### 2.6. ANN dataset and geometry-wise validation

The ANN has five physical inputs, T, W, L, X, and Y, and one output, the surface temperature T_s. Each of the NW, SW, and FW datasets contains 25 geometries, with 846 spatial points per geometry, giving 21,150 samples per cooling state. Inputs and outputs were normalized before training to reduce scale effects. The tested model family was mainly a Multilayer Perceptron (MLP) with one or two hidden layers, including 5-10-1, 5-15-1, 5-20-1, 5-20-10-1, and 5-30-15-1 architectures. For FW, several preprocessing and model variants were tested, while the unseen test geometries were never used for model selection. Dataset structure and ANN geometry-wise validation configuration are shown as in Table 4.

**Table 4:** Dataset structure and ANN geometry-wise validation configuration.

Item	Value	Unit/ detail	Interpretation/ note
ANN inputs	5	T, W, L, X, Y	Spatial surrogate model
ANN output	1	Surface temperature Ts	
Geometry groups	25	Taguchi L25	Geometry is the validation group
Spatial samples per geometry	846	points	All points of one geometry stay together
Samples per cooling condition	21150	points	25 × 846
Cooling conditions	3	NW, SW, FW	
Total samples across conditions	63450	points	3 × 21,150
T input range	0.3–1.1	mm	
W input range	30–40	mm	
L input range	76–96	mm	
X input range	0–130.5	mm	
Y input range	0–88.5	mm	
Primary validation	5-fold grouped cross-validation		No spatial point from a test geometry appears in training
NW/ SW model rule	Final grouped-CV models		Geometry-grouped validation retained
FW model rule	Balanced 15/5/5 train/validation/test within each outer fold	geometry groups	Candidate selection uses validation geometries only; final testing uses unseen geometries
Full-25 model	Retrained after validation		Used for interpolation/ design maps only, not independent validation
Experimental target	Mean of five repetitions		Individual repeat fields were unavailable for pointwise SD/CV reconstruction

Note. All 846 spatial samples belonging to one geometry remain in the same group during validation.

Unlike a random split of individual X-Y points, the validation unit in this study is a complete geometry. In five-fold grouped cross-validation (grouped CV), all 846 points from a test geometry are excluded from training. The final grouped-CV models for NW and SW were locked after the procedure was defined. Because FW showed weaker generalization, its architecture was selected using validation geometries within the training portion; the outer test geometries were used only once for final evaluation. This procedure ensures that no spatial point from a test geometry enters either training or model selection. The Geometry ID-to-fold assignment used in grouped cross-validation is listed in S2 Table in S1 Data.

After geometry-wise evaluation, a model was retrained using all 25 geometries (full-25) for interpolation, response curves, and design maps. The high correlation of the full-25 model reflects interpolation within the known geometry set and is not evidence of independent generalization. The two uses of ANN—prediction of unseen geometries and interpolation within the investigated space—are separated in Fig 10. The corresponding full-data interpolation results for NW, SW, and FW are shown in S4 Fig.

### 2.7. Practical injection-molding demonstration

To show the practical relevance of insert heating to injection molding, an additional experimental dataset from an induction-heated insert platform was used. This is a proof-of-concept test of molding feasibility when the insert surface is heated rapidly before injection. It is not a full controlled experiment designed to isolate the effect of induction heating from all other molding variables. The experimental system and molded part are shown in Fig 19.

In the proof-of-concept test, SABIC 1965T Low-Density Polyethylene (LDPE) was injected at a melt temperature of about 200 °C. The insert was heated for 12 s to a target surface temperature of approximately 210 °C. The high-frequency power was then turned off, the mold was closed, and injection started immediately to limit heat loss. The Chip 2 part was examined during filling and by microscopy. A computer numerical control (CNC) optical measurement system, Nikon NEXIV VMZ-R3020, was used to measure a nominal spacing of 0.030 mm and a nominal height of 0.100 mm. No non-heated control group was available, so these results are interpreted only as a feasibility demonstration and not as a quantitative improvement caused by induction heating.

### 2.8. Performance metrics and statistical analysis

ANN performance was evaluated using the correlation coefficient R, coefficient of determination R², Mean Absolute Error (MAE), Root Mean Square Error (RMSE), Normalized Root Mean Square Error (NRMSE), and mean bias. For N samples, MAE = (1/N)Σ|ŷᵢ − yᵢ| and RMSE = √[(1/N)Σ(ŷᵢ − yᵢ)²]. The residual is defined as ŷᵢ − yᵢ, and the bias is the mean residual. R² was calculated from all out-of-fold predictions in the geometry-wise validation. NRMSE is reported as a percentage of the observed temperature range in the evaluation set.

The effects of T, W, and L were analyzed with the full-25 model after the geometry-wise validation procedure had been locked. These are response analyses within the investigated domain, not causal tests. Because the ANN dataset contains only the mean of five repetitions and not the five individual fields, SD, confidence intervals, and pointwise repeated-measures analysis of variance (ANOVA) were not calculated. The reported statistics are used to assess prediction and design trends; they do not replace uncertainty analysis based on raw repeated measurements.

## 3. Results

### 3.1. Thermal response under different cooling conditions

Cooling state strongly changed both the temperature level and the shape of the thermal field. For T = 0.3 mm, W = 40 mm, and L = 96 mm at t = 5 s, the simulation showed a relatively flat central hot region of about 230 °C for NW, about 150 °C for SW, and only about 45–50 °C for FW (Figs 6 and 7). Thus, water cooling changed not only the maximum temperature but also the temperature gradient and the relative position of the hot region. The large reduction under FW indicates that strong forced convection removes a substantial part of the Joule heat before it can spread across the insert.

**Fig 6:**
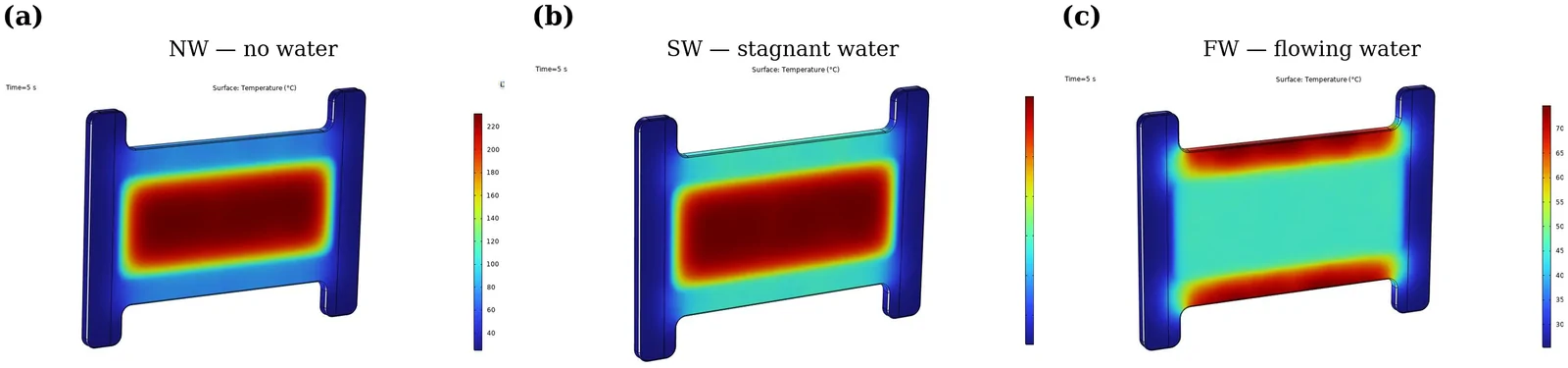
Effect of cooling condition on the simulated temperature field at t = 5 s: (a) no water (NW); (b) stagnant water (SW); (c) flowing water (FW).

**Fig 7:**
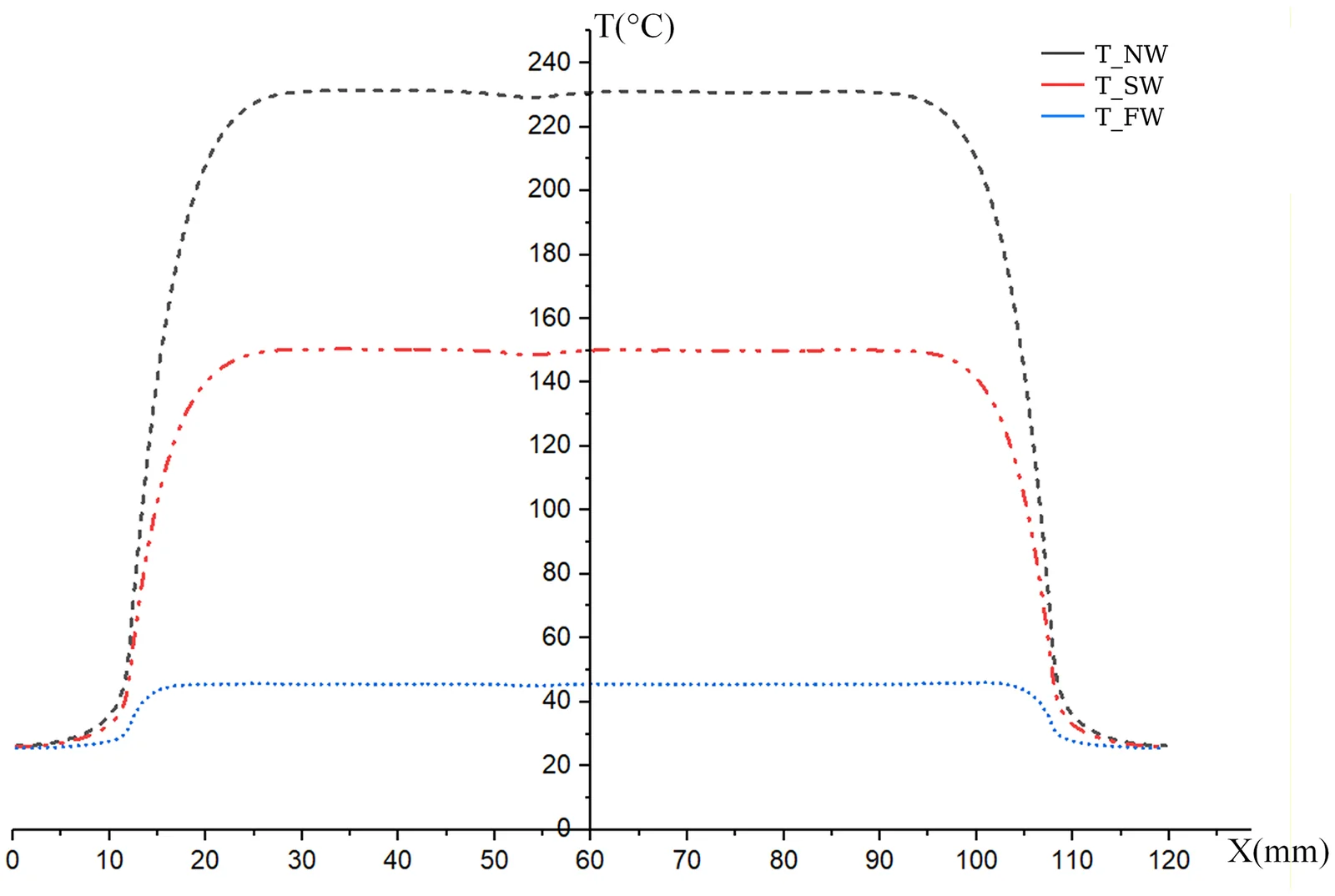
Centerline temperature profiles under the three cooling conditions: no water (NW), stagnant water (SW), and flowing water (FW).

For injection molding, these results show that cooling should be coordinated with the phase of the cycle. NW or weak cooling is suitable during preheating because it allows the cavity surface to reach a high temperature more quickly. After filling and packing, FW can be activated to remove heat before part ejection. If strong FW is maintained during heating, the cavity temperature is suppressed and the benefit of thermal cycling is reduced. The advantage of the structure is therefore not one fixed water state, but the ability to switch the same insert region from heating-dominant to cooling-dominant operation.

### 3.2. Experimental-COMSOL validation

The temperature fields and profiles at t = 5 s show that the numerical model reproduces the location of the hot zone and the overall shape of the experimental distribution (Figs 8 and 9). For T = 0.3 mm, W = 32.5 mm, and L = 91 mm, the simulated peak region was about 180–190 °C, compared with about 160–170 °C in the IR measurement, giving a peak difference of about 20 °C. For representative geometries with T = 0.5, 0.7, and 0.9 mm, the reported differences were no greater than about 10 °C. The T = 1.1 mm case was described as reasonably consistent, but the archived results do not provide a sufficiently clear numerical difference for recalculation. Table 5 therefore summarizes only the evidence available from the source data and does not report global MAE or RMSE. Additional experimental-COMSOL comparisons are shown in S2 Fig and S3 Fig, while the detailed representative-case comparison is provided in S4 Table in S1 Data.

**Table 5:** Summary of COMSOL–experiment validation evidence.

Validation summary item	Value	Interpretation
Representative COMSOL–experiment geometries	5	Five representative cases retained for numerical–experimental validation
Comparison time	5	s
Cases with an explicitly reported numerical temperature difference	4	One case was described qualitatively only
Cases with reported difference ≤ 10 °C	3	T = 0.5, 0.7, and 0.9 mm representative cases
Largest reported peak difference	≈20 °C	T = 0.3 mm, W = 32.5 mm, L = 91 mm

Note. Global COMSOL MAE/RMSE values are not reported because the raw numerical nodal fields were unavailable for re-analysis.

**Fig 8:**
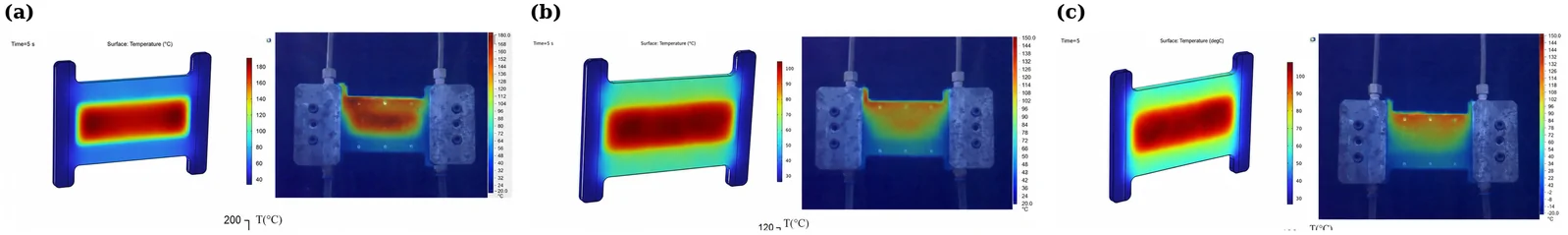
Experimental and simulated temperature fields for three representative insert geometries: (a) T = 0.3 mm, W = 32.5 mm, L = 91 mm; (b) T = 0.7 mm, W = 30 mm, L = 96 mm; (c) T = 1.1 mm, W = 32.5 mm, L = 96 mm. Each panel compares the COMSOL field with the corresponding infrared experimental field at t = 5 s.

**Fig 9:**
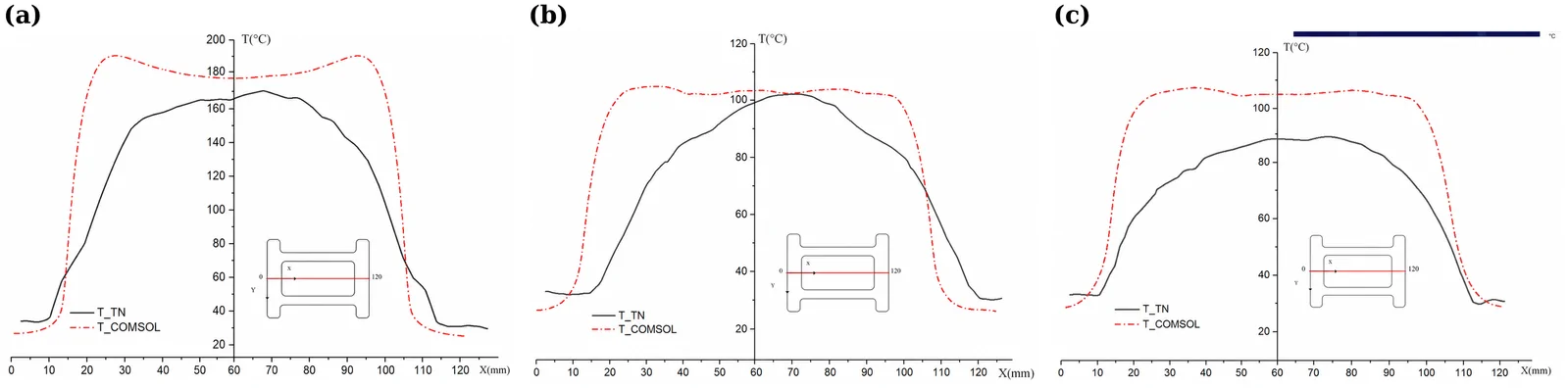
Experimental-COMSOL centerline temperature-profile comparisons for representative insert geometries: (a) T = 0.3 mm, W = 32.5 mm, L = 91 mm; (b) T = 0.7 mm, W = 30 mm, L = 96 mm; (c) T = 1.1 mm, W = 32.5 mm, L = 96 mm.

The numerical error tended to be larger over the high-temperature plateau, while the edge regions and gradient transitions were reproduced more consistently. Possible causes include idealized convection and thermal-contact coefficients, incomplete calibration of temperature-dependent electromagnetic properties, uncertainty in IR emissivity and viewing angle, and differences between the actual clamping condition and the model boundary conditions. A separately calibrated external induction-heating system reported simulation-experiment differences below 5% [16]. The present 10–20 °C differences therefore indicate that the model is more suitable for mechanism interpretation and geometry screening than for absolute temperature metrology.

### 3.3. Geometry-wise ANN validation

Fig 10 summarizes the five-input model and the rule that keeps each complete geometry within a single validation group. The out-of-fold predictions in Figs 11–13 and Table 6 show that NW achieved R = 0.865, R² = 0.746, MAE = 12.56 °C, and RMSE = 17.03 °C; SW achieved R = 0.813, R² = 0.653, MAE = 9.19 °C, and RMSE = 12.97 °C; and FW achieved R = 0.724, R² = 0.493, MAE = 9.33 °C, and RMSE = 12.62 °C. NRMSE was about 11–12% for all three conditions, indicating a similar relative error level despite different temperature ranges. Case-wise geometry-level ANN validation metrics are provided in S3 Table in S1 Data.

**Table 6:** Geometry-wise ANN prediction performance under NW, SW, and FW.

Condition	Validation	R	R²	MAE (°C)	RMSE (°C)	NRMSE (%)	Bias (°C)
NW	Geometry-wise grouped CV (locked)	0.865	0.746	12.56	17.03	11.48	0.96
SW	Geometry-wise grouped CV (locked)	0.813	0.653	9.19	12.97	11.15	0.04
FW	Geometry-wise grouped CV (locked)	0.724	0.493	9.33	12.62	11.65	0.14

Note. NW = No Water; SW = Stagnant Water; FW = Flowing Water.

**Fig 10:**
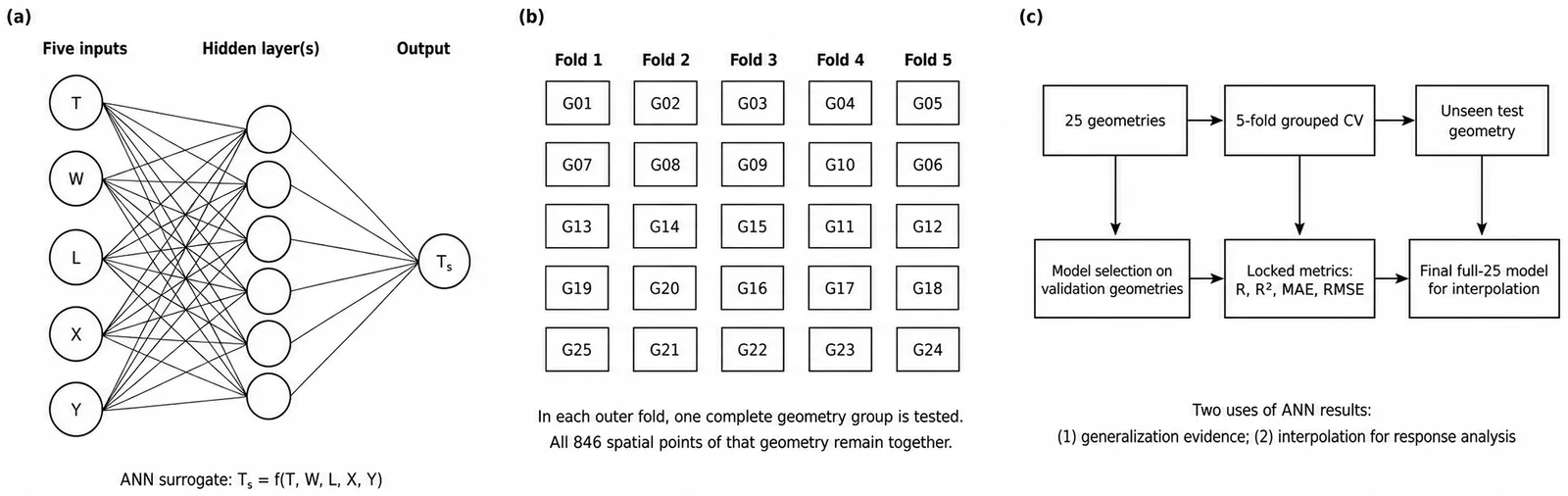
Five-input ANN architecture and geometry-wise validation strategy: (a) ANN surrogate with inputs T, W, L, X, and Y and output surface temperature T_s; (b) allocation of 25 complete geometry groups to five outer folds; (c) workflow separating model selection, unseen-geometry validation, and final full-25 interpolation modeling.

**Fig 11:**
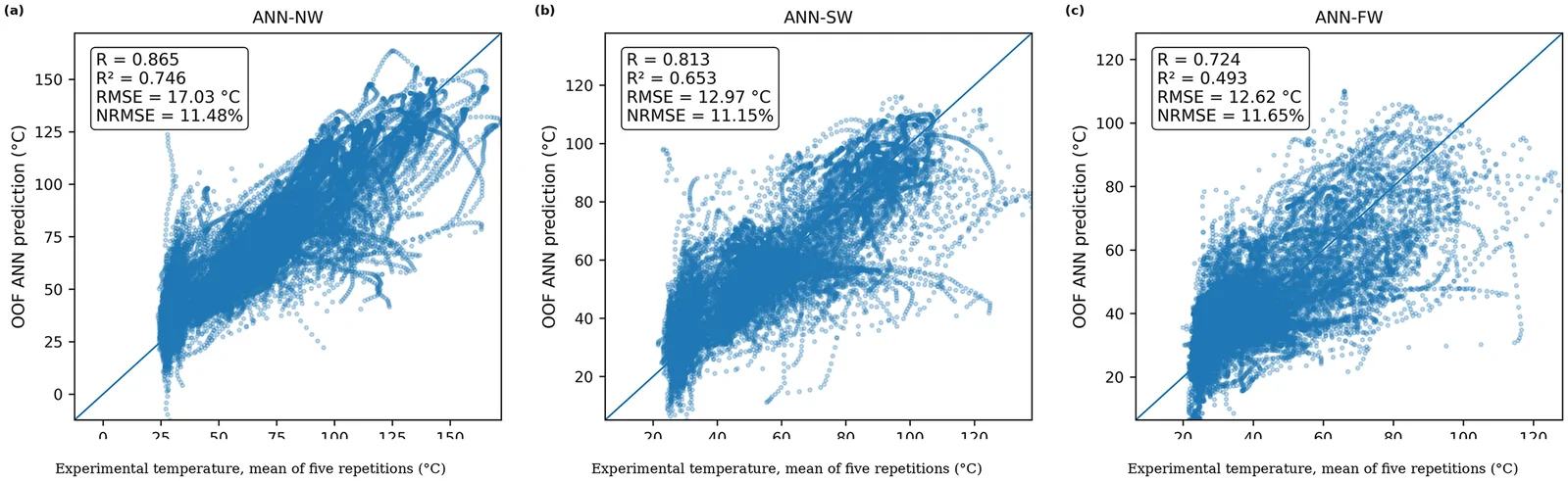
Geometry-wise comparison between ANN predictions and experimental temperatures averaged over five repetitions: (a) no water (NW); (b) stagnant water (SW); (c) flowing water (FW).

**Fig 12:**
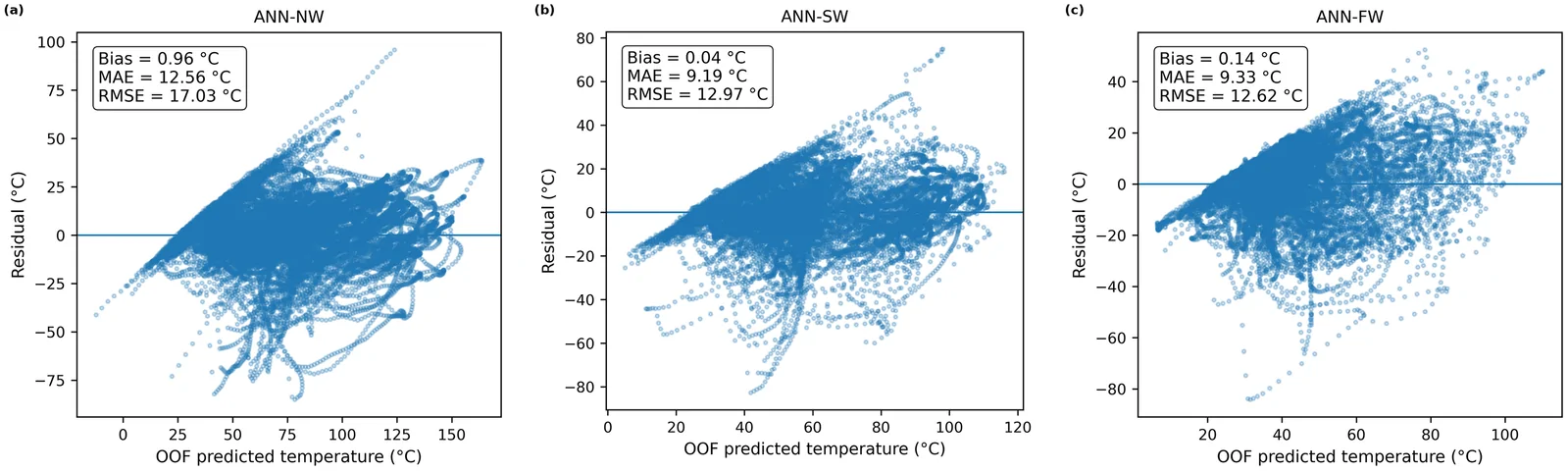
Residual analysis of geometry-wise ANN predictions: (a) no water (NW); (b) stagnant water (SW); (c) flowing water (FW).

**Fig 13:**
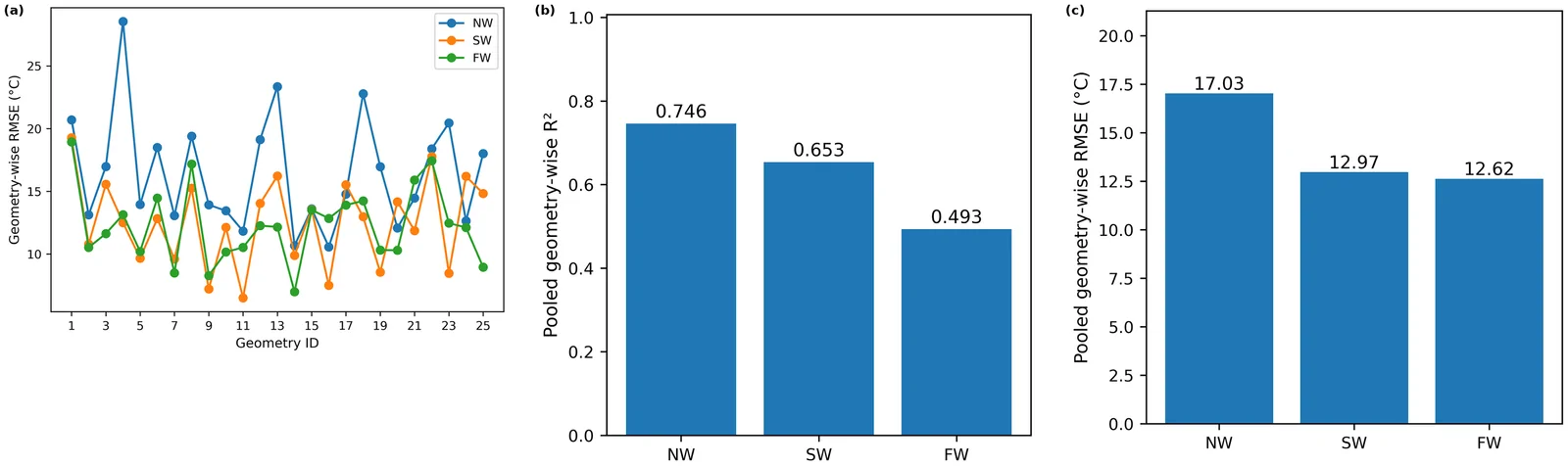
Geometry-wise ANN prediction errors and pooled validation metrics: (a) root mean square error (RMSE) for each Geometry ID under NW, SW, and FW; (b) pooled geometry-wise R² for the three cooling conditions; (c) pooled geometry-wise RMSE for the three cooling conditions.

The mean biases were 0.96, 0.04, and 0.14 °C for NW, SW, and FW, respectively, so the overall average error was close to zero after pooling all unseen geometries. However, several individual geometries still showed large scatter, especially under FW. The optimized FW model, while maintaining strict geometry separation, improved R² from about 0.45 to 0.493 and reduced RMSE from about 13.15 to 12.62 °C. Its performance should still be described as moderate. Under flowing water, the temperature field becomes flatter, so the variation caused by T, W, and L is smaller relative to experimental noise and boundary-condition differences.

Some ANN studies in thermal and material problems report very high R² values when the training and test data are highly similar. For example, greenhouse air-temperature prediction reached R² ≈ 0.997 [20], and a material-strength model reported R² > 0.98 [28]. A mixed-convection problem based on numerical data also reported a correlation coefficient close to 1 [19]. These results are not inconsistent with the present study because the validation question is different: here the ANN must predict a complete geometry that was never used during training. We therefore do not use the full-25 result with R > 0.95 as evidence of independent accuracy. This gives a more realistic estimate of the risk when the surrogate is applied to a new insert geometry.

#### 3.3.1. Effect of insert thickness T.

With W = 35 mm and L = 86 mm fixed, changing T produced a response that depended on cooling state (Fig 14). Under NW, T = 0.3 mm gave the highest centerline peak temperature, about 148.15 °C, with a mean temperature of 94.66 °C. This is consistent with a thinner heating region having lower thermal mass and a different induced-current path. Under SW and FW, however, the highest peaks within the investigated range occurred at T = 0.9 and 0.7 mm, respectively. Convection therefore changes the balance among heat generation, conduction, and heat removal. A simple rule that smaller T is always better should not be applied to all cooling conditions.

**Fig 14:**
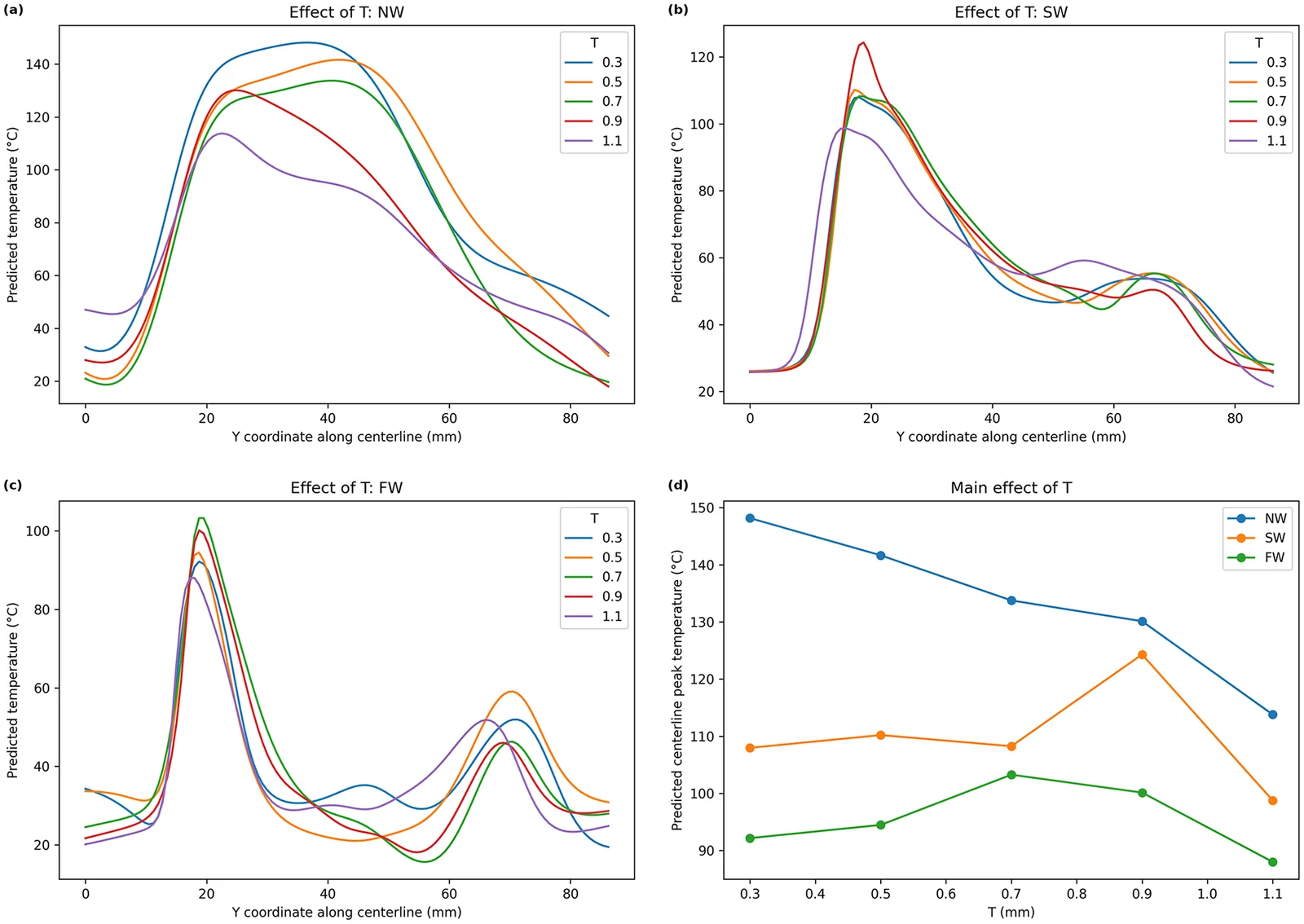
Effect of insert thickness T on the predicted centerline temperature distribution: (a) temperature profiles for no water (NW); (b) temperature profiles for stagnant water (SW); (c) temperature profiles for flowing water (FW); (d) main effect of T on the predicted centerline peak temperature for NW, SW, and FW.

#### 3.3.2. Effect of heating-zone width W.

The effect of W was also non-monotonic across cooling states (Fig 15). Under NW, W = 40 mm gave a centerline maximum of about 140.28 °C and a mean temperature of about 90.89 °C. Under SW, the largest peak in the investigated range occurred at W = 30 mm, whereas FW reached its highest peak at W = 35 mm. Without water, increasing W can enlarge the effective induction-heated area. With convection, that benefit competes with a larger heat-transfer area and a shorter path to the cooling layer. W should therefore be selected together with the cooling strategy.

**Fig 15:**
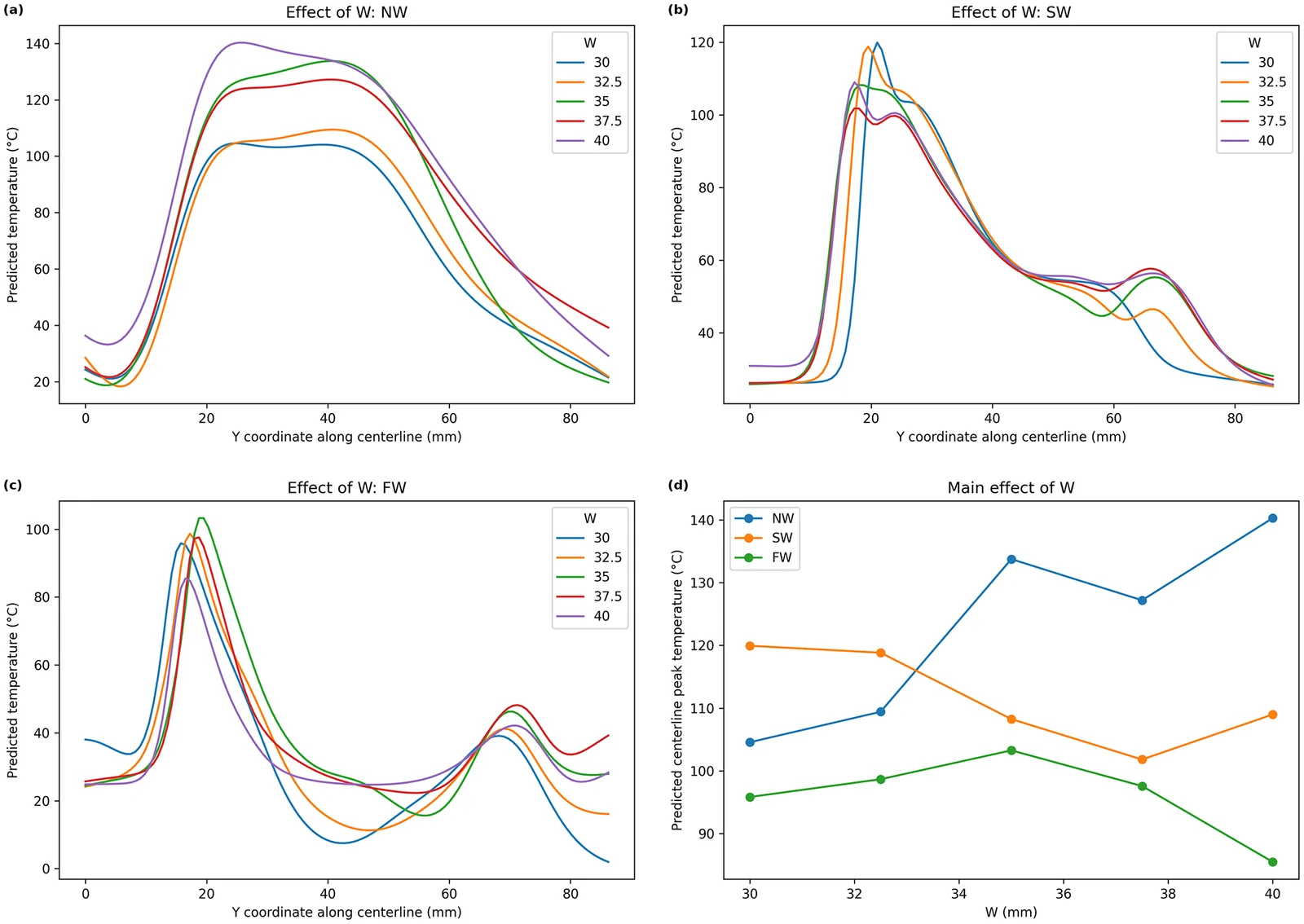
Effect of heating-zone width W on the predicted centerline temperature distribution: (a) temperature profiles for no water (NW); (b) temperature profiles for stagnant water (SW); (c) temperature profiles for flowing water (FW); (d) main effect of W on the predicted centerline peak temperature for NW, SW, and FW.

#### 3.3.3. Effect of heating-zone length L.

With T = 0.7 mm and W = 35 mm, changing L altered both the width of the hot region and the centerline temperature profile (Fig 16). NW reached its highest peak at L = 81 mm, whereas SW and FW both reached their highest peak near L = 86 mm within the investigated range. This shift reflects the interaction among the heated length, longitudinal heat spreading, and heat removal. For injection molding, L should not be selected only from the maximum temperature; the hot region must also cover the required cavity or thin-wall length without creating excessive gradients near the ends.

**Fig 16:**
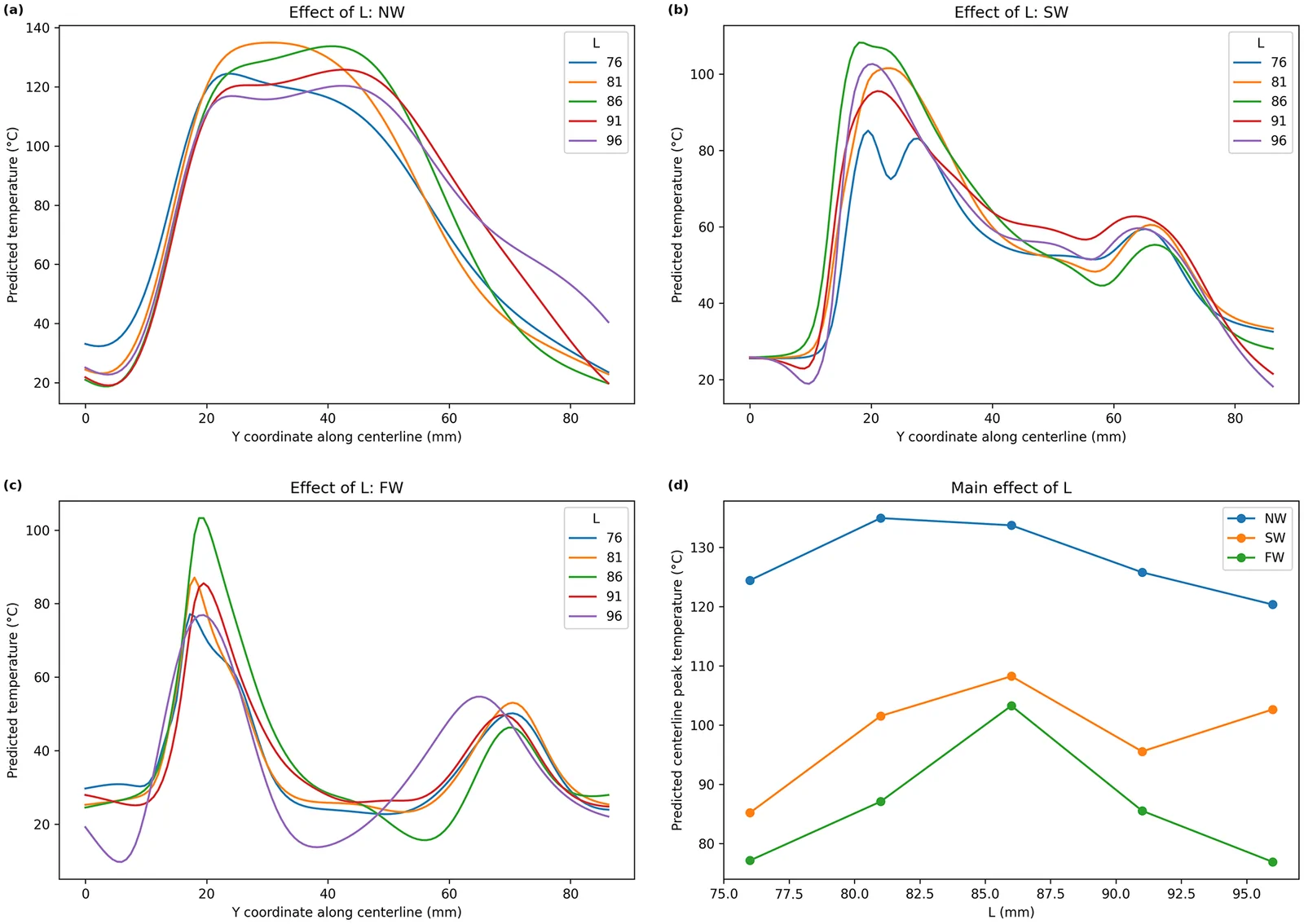
Effect of heating-zone length L on the predicted centerline temperature distribution: (a) temperature profiles for no water (NW); (b) temperature profiles for stagnant water (SW); (c) temperature profiles for flowing water (FW); (d) main effect of L on the predicted centerline peak temperature for NW, SW, and FW.

#### 3.3.4. Effect of cooling condition on the reference geometry.

For the reference geometry T = 0.7 mm, W = 35 mm, and L = 86 mm, the predicted T_max/T_mean/ΔT values were about 133.75/79.58/115.02 °C for NW, 108.25/57.59/82.44 °C for SW, and 103.26/38.47/87.65 °C for FW (Fig 17). T_mean decreased strongly from NW to FW, showing the heat-removal capacity of flowing water. However, ΔT did not decrease monotonically from SW to FW, indicating that strong cooling can create local gradients when the spatial pattern of heat removal does not match the Joule heat-source distribution. In this study, the cooling layer is therefore interpreted mainly as a means to change the thermal state rapidly. A separate benchmark against a conventional cooling channel would be required to quantify any improvement in temperature uniformity.

**Fig 17:**
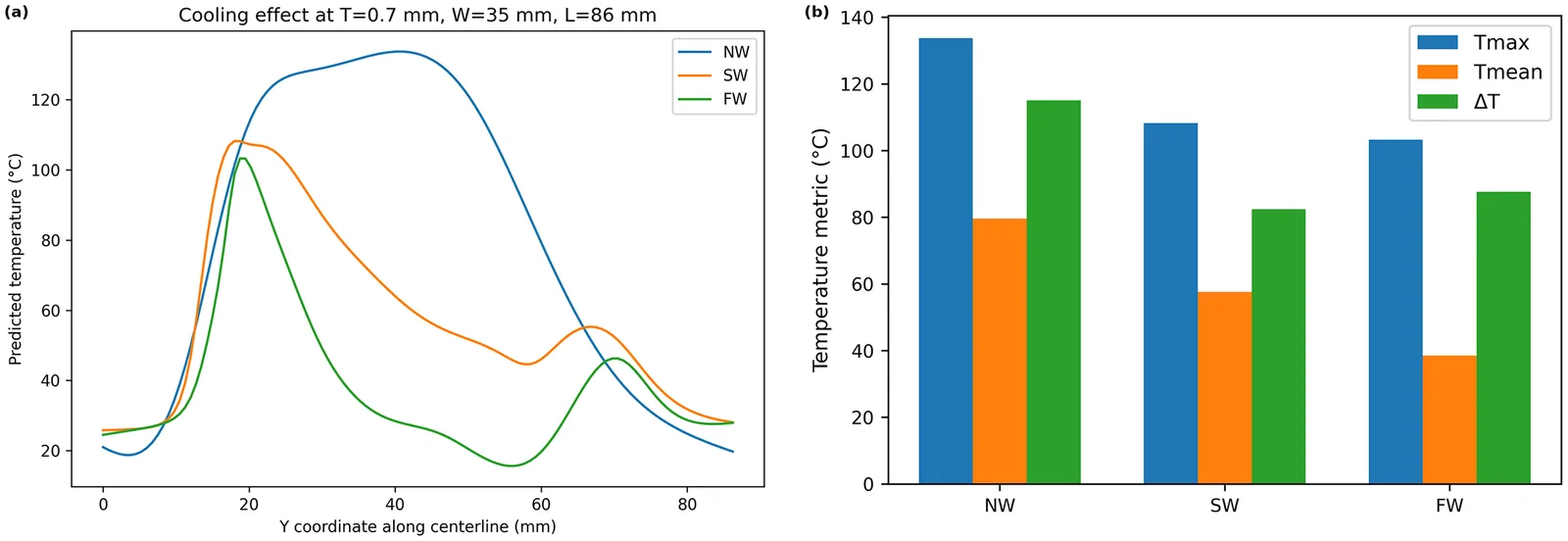
Effect of cooling condition on the thermal response of the reference geometry (T = 0.7 mm, W = 35 mm, L = 86 mm): (a) predicted centerline profiles for NW, SW, and FW; (b) comparison of T_max, T_mean, and ΔT for the three cooling conditions.

#### 3.3.5. Geometry-cooling design maps.

The T-W design maps at L = 86 mm (Fig 18) summarize the geometry-cooling interaction and show that the high-temperature region changes clearly among NW, SW, and FW. NW maintains the highest peak-temperature range and shows strong sensitivity to T and W; SW reduces the range; and FW produces lower peaks and a flatter response. Table 7 summarizes the levels that give the highest centerline peak in each one-factor sweep. These levels are not global optima because the remaining variable is held fixed, but they are useful for screening designs before additional experiments or simulations.

**Table 7:** ANN-derived factor effects and cooling-response summary within the investigated design space.

Analysis	Condition	Level/ reference geometry	Tmax (°C)	Tmean (°C)	ΔT (°C)	Explanation
Thickness T sweep	NW	T = 0.3 mm	148.15	94.66	116.75	Highest centerline peak in the NW T sweep
	SW	T = 0.9 mm	124.29	57.07	98.45	Non-monotonic response under stagnant water
	FW	T = 0.7 mm	103.26	38.47	87.65	Non-monotonic response under flowing water
Width W sweep	NW	W = 40 mm	140.28	90.89	111.07	Highest centerline peak in the NW W sweep
	SW	W = 30 mm	119.93	52.76	94.18	Cooling changes the apparent main-effect trend
	FW	W = 35 mm	103.26	38.47	87.65	Peak response occurs at an intermediate width
Length L sweep	NW	L = 81 mm	134.97	78.74	112.11	Highest centerline peak in the NW L sweep
	SW	L = 86 mm	108.25	57.59	82.44	Intermediate length gives the highest peak
	FW	L = 86 mm	103.26	38.47	87.65	Intermediate length gives the highest peak
Cooling response	NW	T = 0.7, W = 35, L = 86 mm	133.75	79.58	115.02	Reference geometry without water cooling
	SW	T = 0.7, W = 35, L = 86 mm	108.25	57.59	82.44	Stagnant-water cooling reduces thermal level
	FW	T = 0.7, W = 35, L = 86 mm	103.26	38.47	87.65	Flowing-water cooling gives the lowest mean temperature

Note. For each one-factor sweep, the row reports the level giving the highest predicted centerline peak temperature; this is not a global optimum.

**Fig 18:**
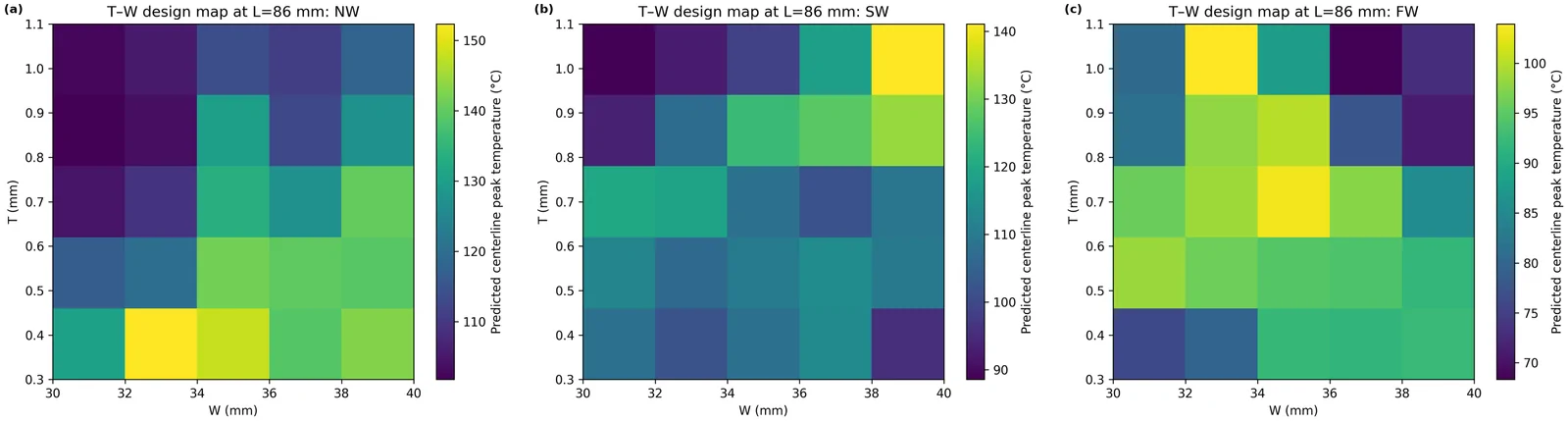
Geometry-cooling design maps for the induction self-heating insert at L = 86 mm: (a) T-W map for no water (NW); (b) T-W map for stagnant water (SW); (c) T-W map for flowing water (FW).

From a mold-design perspective, the maps support two operating strategies. If filling is the priority during injection, a geometry with a high thermal response under NW or SW can be selected and FW can be activated only after filling. If thermal stability or a temperature-sensitive polymer is the priority, a moderate-response region can be selected and FW can start earlier. This is the practical value of the surrogate model: rather than returning one ‘best’ geometry, it supports trade-offs among peak temperature, mean temperature, temperature gradient, and cooling capacity.

### 3.4. Practical molding demonstration of the proposed structure

Fig 19 provides additional application evidence from an induction-heated mold platform. For LDPE, after 12 s of heating to a target of about 210 °C, the Chip 2 part filled from the central region through the connecting section and then toward both ends until the geometry was completely filled. The experimental images show no large short-shot region, and the main holes and connecting features were formed clearly. Microscopy of three parts shows that the series of circular microfeatures was replicated and remained distinguishable, although the LDPE surfaces showed more texture than some amorphous polymers.

**Fig 19:**
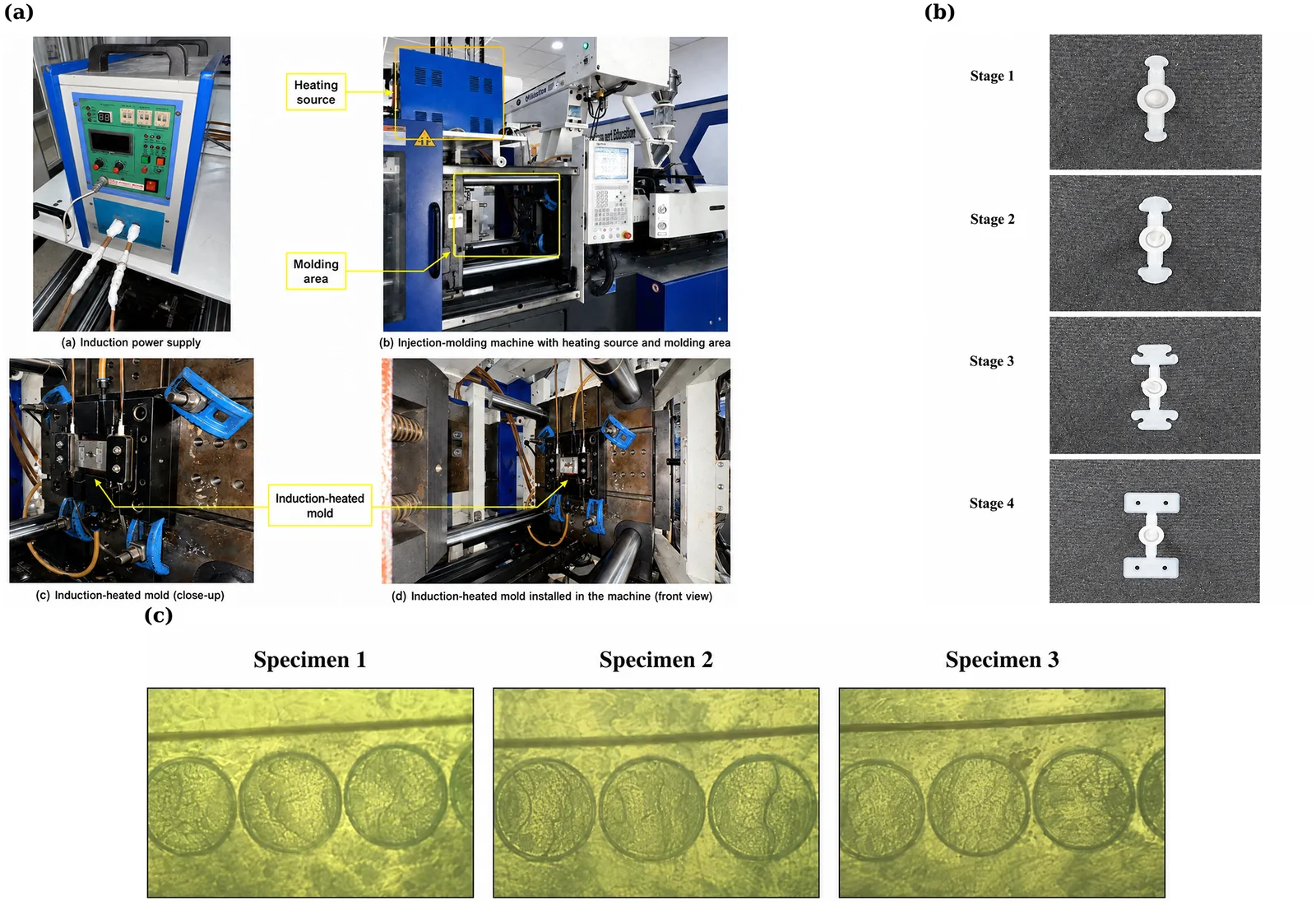
Practical molding demonstration of the induction-heating structure: (a) experimental injection-molding system and induction-heated mold; (b) four-stage LDPE Chip 2 filling progression from partial filling to complete filling; (c) microscopic replication of circular microfeatures in three LDPE specimens.

Optical measurement of the LDPE parts gave a mean spacing of 28.6 µm for a nominal 30 µm and a mean height of 106.6 µm for a nominal 100 µm; the two individual height measurements were 105.7 and 107.5 µm. These results show that the high-temperature window before injection was sufficient to maintain flow into small features. However, because no non-heated control group was available and the additional molding platform differs from the 25 main self-heating geometries, Fig 19 is treated only as proof of feasibility. It is not quantitative evidence that the main structure improves filling by a specific amount.

## 4. Discussion

### 4.1. Physical interpretation of induction self-heating

The key physical feature of the insert is the combination of local electromagnetic heat generation, a thin heating region, and a short heat-transfer path. A previous induction-heating study reported a heating rate of about 22.5 °C/s as the mold surface increased from 110 to 200 °C in 4 s [11]. For a thin-wall product, a recent external induction configuration showed that a 5–8 s heating window was sufficient to improve flow conditions and mechanical performance [16]. The present study also produced a clear hot region after 5 s, placing its response in the same rapid-heating range. The main difference is that the temperature field is analyzed together with T-W-L geometry and three cooling-water states rather than only during the heating stage.

The benefit to injection molding follows directly from the thermal state at the mold-polymer interface. A hotter cavity surface reduces heat loss from the near-wall polymer, delays frozen-layer formation, and keeps a larger effective flow area. This can increase flow length, reduce short shots in thin or remote regions, and improve replication of small features [2,3]. A higher surface temperature also gives the two flow fronts at a weld line more time to contact and interdiffuse before solidification, which can improve appearance and bonding [10,11]. Because only the functional insert region is heated rather than the whole mold base, the thermal mass that must be raised in temperature is smaller. When a near-surface cooling layer is added, the same region can switch quickly from filling-oriented heating to solidification-oriented cooling.

### 4.2. Effects of insert geometry and cooling condition

T, W, and L do not act independently. T changes the thermal mass, the induced-current path, and the conduction path at the same time. W and L change both the heat-generation area and the heat-transfer area to colder regions. Under NW, Joule heating dominates and the effect of geometry is more evident. Under SW and especially FW, heat removal increases and the response becomes the result of two competing fields: electromagnetic heat generation and conduction-convection cooling. This explains why the highest temperatures under SW and FW can occur at intermediate factor levels instead of always at the smallest T or the largest W and L.

This result is important for design because it treats cooling state as a variable alongside geometry rather than examining only the heating stage. Many induction-heating studies focus on coil distance, coil geometry, or heating uniformity [6,36,38], while hot-gas and infrared systems mainly aim to raise the cavity temperature rapidly before filling [14,41,42]. Cooling-layer studies, in contrast, show that a coolant layer placed close to the cavity can increase heat-removal rate and reduce temperature variation [45,46]. One conformal cooling-layer design reduced cooling time by 26.34% compared with conventional drilled channels [47]. The present study does not directly benchmark cycle time against a conventional mold, but the reduction of the simulated thermal plateau from about 230 °C under NW to about 45–50 °C under FW shows that water state has a strong effect on the thermal field. A thin heating region directly adjacent to the water layer therefore provides a short heat-transfer path during both heating and cooling, which is structurally useful for injection-molding cycles.

### 4.3. Reliability and limitations of the numerical model

The numerical model reproduced the hot region reasonably well, but agreement was not uniform across all geometries. One case showed a peak difference of nearly 20 °C, while several others were within about 10 °C. The model should therefore be interpreted as a mechanism and design-screening model rather than as a metrologically calibrated temperature predictor. This limitation is consistent with the modeling assumptions recovered from the archived COMSOL files: the C45 insert was represented by a constant soft-iron surrogate property set, temperature dependence of electrical and thermal properties was not included, solid-solid thermal contact resistance was neglected, and the cooling-water states were represented by prescribed convection coefficients rather than a resolved fluid-flow model. A formal mesh-independence study and a separate time-step sensitivity study were also not retained, so the present work does not claim mesh- or time-step-independent absolute temperatures.

Even with these limits, the model remains useful for mechanism interpretation because the hot-zone location, gradient regions, and cooling effects are reproduced consistently. The retained free-tetrahedral mesh and adaptive BDF solver settings are now reported explicitly to improve reproducibility. In mold design, this model can be used to reject unsuitable geometries before fabrication, provided that absolute temperatures are checked by infrared measurement or contact sensors. Future work should include temperature-dependent C45 electromagnetic and thermal properties, measured electrical/thermal contact resistance, experimentally characterized water flow and inlet temperature, formal mesh and temporal sensitivity studies, and retained nodal fields for spatially matched MAE/RMSE calculations.

### 4.4. ANN generalization and interpolation

The ANN results highlight the difference between interpolation and generalization. If the 21,150 points in one cooling state are split randomly by spatial point, samples from the same geometry appear in both training and test sets and the correlation can become very high. In this study, the full-25 model achieved R > 0.95 for interpolation, but this value is used only for response analysis after validation is complete. When the ANN is required to predict a completely unseen geometry, R² decreases to 0.746, 0.653, and 0.493 for NW, SW, and FW. This is a stricter test, but it is more relevant to the practical question of predicting a new insert geometry.

The lower R² values should be interpreted together with the data-splitting rule. An MLP model for greenhouse temperature reported R² ≈ 0.997 and RMSE = 0.439 °C when tested on repeated time-series measurements from the same physical system [20]. A material-strength network reported R² > 0.98 on a limited dataset [28], while an ANN for mixed convection based on numerical data reported a correlation coefficient close to 1 [19]. These examples show that ANN models can fit very closely when the training and test data occupy nearby parts of the same data domain. In contrast, the present geometry-wise split deliberately separates training and testing at the geometry level. The resulting R² is lower, but it better answers whether a new insert can be predicted. Studies that combine ANN with optimization also support keeping model selection separate from the final test set to reduce overfitting [27].

FW is the most difficult case because strong cooling reduces the temperature range and the sensitivity of temperature to T, W, and L. When the geometry-related signal becomes smaller, variation from boundary conditions, the camera, and assembly has a larger relative effect. This explains why architecture optimization improved FW R² only from about 0.45 to 0.493. Rather than increasing the number of neurons to chase test performance, the model was locked at this stage and FW is treated as a moderate-prediction case. Further improvement should come from more geometries or physical inputs describing water flow rate and temperature, not only from a more complex ANN.

### 4.5. Practical implications and study limitations

For injection molding, the main practical advantage of the structure is the ability to create a high-temperature window just before and during filling and then switch rapidly to cooling. During injection, the hot surface delays frozen-layer formation, preserves the effective flow area, and improves filling of thin walls or microfeatures [2,7]. At weld lines, a longer contact time at high temperature can improve bonding and appearance [10,17]. After filling and packing, the cooling layer close to the thin insert provides a short path for removing heat before ejection. Near-cavity cooling layers and conformal cooling have shown benefits in heat-removal rate and cycle-time reduction [45,47]. The structure therefore addresses two opposing needs of injection molding within the same low-thermal-mass functional region: a hot cavity when flow is required and a cool cavity when ejection is required.

This study has several limitations. First, the ANN dataset stores only the mean of five repetitions, so between-run uncertainty cannot be quantified from the retained ANN dataset. Second, the numerical model uses a constant soft-iron surrogate property set for the C45 insert, prescribed cooling convection, perfect thermal continuity at solid interfaces, and insulated unassigned exterior boundaries; formal mesh-independence and time-step sensitivity studies were not retained. Third, complete nodal fields were not retained for all numerical cases, so global full-field validation metrics cannot be recalculated. Fourth, the molding demonstration has no non-heated control group; complete filling therefore cannot be attributed quantitatively to induction heating alone. Fifth, the additional molding platform does not exactly match the 25 main self-heating geometries. Finally, electrical-to-thermal efficiency was not measured directly, and the cooling layer was not benchmarked against a conventional cooling-channel system. Claims about heating and cooling efficiency are therefore limited to the structural mechanism and the available thermal data. Future work should include controlled comparisons, raw repeated measurements, electrical and thermal power measurements, more polymer materials, and experimentally measured water-flow and water-temperature inputs.

## 5. Conclusions

This study developed an integrated evaluation framework for an induction self-heating insert using a Taguchi L25 design, IR temperature measurements, a sequentially coupled electromagnetic-thermal model, and a coordinate-based ANN. The results show that T-W-L geometry and cooling state jointly determine the thermal field. Strong cooling can substantially reduce temperature and can change the apparent ranking of geometric effects, so no single one-factor rule applies to all cooling states. The numerical model reproduced the hot-zone location and the main temperature-profile trends at t = 5 s. Most reported comparison cases differed by about 10 °C or less, although one case differed by about 20 °C, indicating the need for better calibration of material properties and boundary conditions.

Geometry-wise validation gives a more conservative ANN assessment than a random split of spatial points. NW, SW, and FW achieved R² values of 0.746, 0.653, and 0.493, with RMSE values of 17.03, 12.97, and 12.62 °C, respectively. FW showed weaker generalization because forced convection flattened the thermal field, although the mean bias remained close to zero. The full-25 models were used only for interpolation, response curves, and design maps after the unseen-geometry validation procedure had been locked.

For injection molding, the thin heating region is integrated into the induction-current path and placed close to the cooling layer, giving a short energy-transfer path during both heating and cooling. During injection, a hotter cavity can delay frozen-layer formation, support flow into thin-wall or microstructured regions, and improve weld-line conditions. After filling, flowing water can remove heat before ejection. The LDPE proof-of-concept test produced a completely filled Chip 2 part with replicated small features. The mean spacing was 28.6 µm and the mean height was 106.6 µm, close to the nominal 30 and 100 µm dimensions. This demonstrates technical feasibility, but it does not replace a controlled heated/non-heated comparison or a full cooling-channel benchmark.

Overall, the study moves beyond simply maximizing mold temperature toward a combined design of the self-heating region, cooling state, and ANN validation on unseen geometries. The approach provides a basis for selecting insert geometry for a specific molding objective, switching water conditions across the cycle, and using the surrogate model as a screening tool before fabrication or more detailed simulation.

## Supporting information

S1 Fig Infrared thermal measurement system: (a) SmartView thermal-analysis software interface; (b) infrared thermal camera used in the experiments. (TIF)

S2 Fig Additional experimental-COMSOL comparison for representative geometry I, including the simulated temperature field, the corresponding experimental infrared temperature field, and the experimental-COMSOL centerline temperature profiles. (TIF)

S3 Fig Additional experimental-COMSOL comparison for representative geometry II, including the simulated temperature field, the corresponding experimental infrared temperature field, and the experimental-COMSOL centerline temperature profiles. (TIF)

S4 Fig Full-data interpolation performance of the final ANN models: (a) ANN-NW; (b) ANN-SW; (c) ANN-FW. These plots describe interpolation after full-25 retraining and are not used as independent geometry-wise validation.
(TIF)

S1 Data Include: S1 Table. Complete Taguchi L25 geometry matrix; S2 Table. Geometry ID and grouped cross-validation fold assignment; S3 Table. Case-wise geometry-level ANN validation metrics; S4 Table. Detailed COMSOL-experiment comparison for representative geometries. (XLSX)

S1 File ANN. (ZIP)
