# Supplementary material for: Geometry and cooling effects on an induction self-heating insert for rapid mold temperature control: Experimental, Multiphysics, and artificial neural network-based analysis
Source: PLoS One. 2026 Oct 27;21(10):e0359849. doi: 10.1371/journal.pone.0359849 (PMC13712609; doi:10.1371/journal.pone.0359849)
Supplement: Supplementary file 1 [file pone.0359849.s008.docx]

**Response to Reviewer #3**

*Geometry and Cooling Effects on an Induction Self-Heating Insert for Rapid Mold Temperature Control: Experimental, Multiphysics, and Artificial Neural Network-Based Analysis*

**Dear Reviewer#3,**

We thank you very much for the concise recommendations. The revision reduces grouped citations, emphasizes recent literature, clarifies experimental uncertainty and ANN limitations, expands the archived COMSOL formulation and solver details, and narrows the molding/cooling claims where a control experiment is not available.

## Comment 1

**Reviewer comment:** *Avoid multiple paragraph breakage and multiple citations at a time. Please cite single sentence with a single reference only.*

**Response:** We revised the literature-heavy paragraphs so that individual claims are supported by a single reference per sentence rather than grouped citation blocks. We also consolidated related discussion to reduce unnecessary paragraph fragmentation.

**Changes in the revised manuscript:** The Introduction and Discussion literature paragraphs were rewritten throughout.

## Comment 2

**Reviewer comment:** *Most of the cited reference are outdated. Please provide the recent citation of 2024-2026.*

**Response:** We retained a limited number of older references where they are foundational to induction heating and variotherm molding, but increased the use of recent literature. In addition to the existing 2024–2026 references, we added two 2026 studies suggested by Reviewer #1 and a 2025 machine-learning review.

**Changes in the revised manuscript:** Refs. 49–51 were added and the ANN literature discussion was updated.

## Comment 3

**Reviewer comment:** *Please report experimental uncertainty more clearly.*

**Response:** We now distinguish the IR camera manufacturer-specified accuracy (approximately ±2 °C) from experimental repeatability. Because only the mean of five thermal repeats is retained, pointwise repeatability SD/error bars cannot be reconstructed. For the Chip 2 dimensional measurements, feature-level data are available and we now report n and sample SD.

**Changes in the revised manuscript:** Sections 2.2, 2.7, 2.8, 3.4, and 4.5 were revised.

## Comment 4

**Reviewer comment:** *Provide stronger numerical model validation, the current form is not sufficient.*

**Response:** We strengthened the numerical section by reporting the actual archived multiphysics formulation, material properties, mesh settings, solver settings, cooling boundaries, and model assumptions. We also retain five representative experiment–COMSOL cases and additional comparisons in S2 and S3 Figs. However, full nodal fields are not available for all cases, so we do not introduce a global MAE/RMSE that cannot be reproduced. The model is now explicitly described as suitable for mechanism interpretation and design screening rather than metrological temperature prediction.

**Changes in the revised manuscript:** Sections 2.4, 3.2, 4.3, Table 2, Table 5, S2 Fig, S3 Fig, and S4 Table are referenced more explicitly.

## Comment 5

**Reviewer comment:** *Clarify the ANN performance under flowing water.*

**Response:** We agree. FW is now explicitly described as a moderate-prediction case (R² = 0.493), not a highly predictive model. We explain that forced cooling flattens the temperature field and reduces the geometry-related signal relative to experimental/boundary variability.

**Changes in the revised manuscript:** Sections 3.3 and 4.4 were revised.

## Comment 6

**Reviewer comment:** *Include a non-heated control for the molding test.*

**Response:** We agree that a non-heated control would be required for a causal comparison. Such a control was not included in the retained molding dataset and cannot be reconstructed retrospectively. We therefore revised the manuscript so that Fig. 19 is explicitly a proof-of-concept feasibility demonstration only and does not claim an improvement relative to non-heated molding.

**Changes in the revised manuscript:** Sections 2.7, 3.4, 4.5, the Abstract, and the Conclusion were revised.

## Comment 7

**Reviewer comment:** *Discuss the ANN’s applicability beyond the tested geometries.*

**Response:** We now state the validated applicability domain explicitly: T = 0.3–1.1 mm, W = 30–40 mm, L = 76–96 mm, X = 0–130.5 mm, and Y = 0–88.5 mm under NW, SW, and FW. The full-25 models are described as interpolation/screening tools within this domain. Predictions outside these ranges are extrapolations and were not validated.

**Changes in the revised manuscript:** Section 4.4 was expanded.

## Comment 8

**Reviewer comment:** *Compare the proposed cooling design with conventional channels.*

**Response:** We agree that a direct comparison with conventional straight channels would be useful. The current dataset does not include a matched conventional-channel benchmark. We therefore do not claim a quantified improvement in cooling time or uniformity over conventional channels. The discussion uses prior cooling-layer/conformal-cooling literature only as context, and the absence of a direct benchmark is now stated explicitly as a limitation.

**Changes in the revised manuscript:** Sections 3.3.4, 4.2, 4.5, and the Conclusion were revised.

We appreciate your insightful comments, which have substantially improved the manuscript’s clarity and rigor. We attach the revised version (clean and tracked) together with this response letter.

***With best regards,***

On behalf of all co-authors

Faculty of Mechanical Engineering, HCM-UTE
