# Supplementary material for: Geometry and cooling effects on an induction self-heating insert for rapid mold temperature control: Experimental, Multiphysics, and artificial neural network-based analysis
Source: PLoS One. 2026 Oct 27;21(10):e0359849. doi: 10.1371/journal.pone.0359849 (PMC13712609; doi:10.1371/journal.pone.0359849)
Supplement: Peer review history

# Peer review: Decision Letter 0

**DOI:** 10.1371/journal.pone.0359849.r001

**Authors:** Gaurav Arora

29 Aug 2026

PLOS One

Dear Dr. Son Minh,

Thank you for submitting your manuscript to PLOS One. After careful consideration, we feel that it has merit but does not fully meet PLOS One’s publication criteria as it currently stands. Therefore, we invite you to submit a revised version of the manuscript that addresses the points raised during the review process.

**ACADEMIC EDITOR:**

Dear Authors,

Thank you for submitting your manuscript. Your paper has now been evaluated by our expert reviewers, and while they recognized the potential value and relevance of your research, they raised substantial concerns. Consequently, I invite you to submit a substantially revised version of your manuscript that addresses all appended reviewer comments in full; please note that because this is a Major Revision request, acceptance is not guaranteed and the paper will likely undergo a second round of external peer review. When preparing your resubmission, which is requested by the deadline mentioned, please pay special attention to ensuring that your methods are fully replicable, your conclusions do not overreach the data, and that you include a detailed, point-by-point "Response to Reviewers" document highlighting exactly where changes were made in the text.

Please submit your revised manuscript by Oct 28 2026 11:59PM. If you will need more time than this to complete your revisions, please reply to this message or contact the journal office at plosone@plos.org. When you're ready to submit your revision, log on to https://www.editorialmanager.com/pone/ and select the 'Submissions Needing Revision' folder to locate your manuscript file.

- A letter that responds to each point raised by the academic editor and reviewer(s). You should upload this letter as a separate file labeled 'Response to Reviewers'.
- A marked-up copy of your manuscript that highlights changes made to the original version. You should upload this as a separate file labeled 'Revised Manuscript with Track Changes'.
- An unmarked version of your revised paper without tracked changes. You should upload this as a separate file labeled 'Manuscript'.

If you would like to make changes to your financial disclosure, please include your updated statement in your cover letter. Guidelines for resubmitting your figure files are available below the reviewer comments at the end of this letter.

If applicable, we recommend that you deposit your laboratory protocols in protocols.io to enhance the reproducibility of your results. Protocols.io assigns your protocol its own identifier (DOI) so that it can be cited independently in the future. For instructions see: https://journals.plos.org/plosone/s/submission-guidelines#loc-laboratory-protocols. Additionally, PLOS One offers an option for publishing peer-reviewed Lab Protocol articles, which describe protocols hosted on protocols.io. Read more information on sharing protocols at https://plos.org/protocols?utm_medium=editorial-email&utm_source=authorletters&utm_campaign=protocols.

As the corresponding author, your ORCID iD is verified in the submission system and will appear in the published article. PLOS supports the use of ORCID, and we encourage all coauthors to register for an ORCID iD and use it as well. Please encourage your coauthors to verify their ORCID iD within the submission system before final acceptance, as unverified ORCID iDs will not appear in the published article. *Only*  the individual author can complete the verification step; PLOS staff *cannot*  verify ORCID iDs on behalf of authors.

We look forward to receiving your revised manuscript.

Kind regards,

Academic Editor

PLOS One

Journal Requirements:

If the reviewer comments include a recommendation to cite specific previously published works, please review and evaluate these publications to determine whether they are relevant and should be cited. There is no requirement to cite these works unless the editor has indicated otherwise.

Additional Editor Comments:

Dear Authors,

Thank you for submitting your manuscript. Your paper has now been evaluated by our expert reviewers, and while they recognized the potential value and relevance of your research, they raised substantial concerns. Consequently, I invite you to submit a substantially revised version of your manuscript that addresses all appended reviewer comments in full; please note that because this is a Major Revision request, acceptance is not guaranteed and the paper will likely undergo a second round of external peer review. When preparing your resubmission, which is requested by the deadline mentioned, please pay special attention to ensuring that your methods are fully replicable, your conclusions do not overreach the data, and that you include a detailed, point-by-point "Response to Reviewers" document highlighting exactly where changes were made in the text.

[Note: HTML markup is below. Please do not edit.]

Reviewers' comments:

Reviewer's Responses to Questions

**Comments to the Author**

1. Is the manuscript technically sound, and do the data support the conclusions?

Reviewer #1: Partly

Reviewer #2: Yes

Reviewer #3: Yes

**********

2. Has the statistical analysis been performed appropriately and rigorously?

Reviewer #1: Yes

Reviewer #2: Yes

Reviewer #3: Yes

**********

3. Have the authors made all data underlying the findings in their manuscript fully available?

The PLOS Data policy

Reviewer #1: Yes

Reviewer #2: Yes

Reviewer #3: Yes

**********

4. Is the manuscript presented in an intelligible fashion and written in standard English?

Reviewer #1: No

Reviewer #2: Yes

Reviewer #3: Yes

**********

Reviewer #1: The study is technically promising, but stronger experimental validation, detailed multiphysics formulation, ANN methodology, uncertainty analysis, energy/cycle-time assessment, and controlled molding comparisons are required to substantiate the proposed thermal-control strategy

1. The electromagnetic–thermal model should provide the governing equations, mesh size, time-step sensitivity, convergence criteria, and temperature-dependent electrical/thermal properties used in the simulation.

2. The Taguchi L25 design should be presented explicitly, including the actual levels of T, W, and L, their physical meaning, and the rationale for selecting these ranges.

3. The effects of cooling conditions (NW, SW, and FW) require quantitative analysis. Report water temperature, flow rate, Reynolds number, cooling-channel dimensions, and convective heat-transfer coefficients.

4. Since the experimental ANN targets are based on the mean of five repeated measurements, the manuscript should report standard deviation/error bars and assess the repeatability and uncertainty of the temperature measurements.

5. The reported ANN performance is relatively moderate, particularly R² = 0.493 for flowing water. The manuscript should avoid presenting the ANN as a highly predictive model and should discuss its limitations for design optimization.

6. These studies can be helpful for this work:

a. Computational analysis of polymer carbon nanotube composites using experiments and ANN modeling for manufacturing applications

b. Investigation of tribological behaviour of B4C–graphene glass fiber composites using Taguchi, multi-criteria decision making, and machine learning approaches

7. A sensitivity/ablation analysis should quantify the individual and interaction effects of T, W, L, X, Y, and cooling condition on predicted temperature.

8. The proof-of-concept result of 28.6 µm spacing versus 30 µm nominal and 106.6 µm height versus 100 µm nominal should include measurement uncertainty, number of measured features, standard deviation, and measurement technique.

9. The manuscript should quantify whether induction heating actually reduces cycle time, filling pressure, injection pressure, or frozen-layer thickness compared with conventional mold-temperature control.

Reviewer #2: 1. The authors should provide a more rigorous quantitative analysis linking the processing routes (MA–HPS and VAM) with phase evolution, grain structure, porosity, and elemental segregation, rather than relying primarily on qualitative SEM observations.

2. The reported grain sizes of 25.1 nm for HPS and 46 nm for VAM require careful verification, since such dimensions should be supported by a clearly described measurement methodology, sufficient sampling statistics, and preferably complementary XRD-based crystallite-size analysis.

3. The phase identification of the BCC matrix and Nb₅Si₃ should be strengthened by providing quantitative phase fractions and detailed XRD analysis, because the subsequent explanations of precipitation strengthening, grain-boundary strengthening, and silicide effects depend strongly on the actual amount and distribution of these phases.

4. The mechanical-property comparison should include repeated measurements, standard deviations or error bars, and statistical significance, particularly for the reported yield strength, ultimate compressive strength, fracture strain, hardness, and wear rate, to establish whether the differences between HPS and VAM are statistically meaningful.

5. The corrosion analysis should provide sufficient electrochemical testing details, including exposed surface area, electrolyte preparation, stabilization time, scan rate, fitting procedure, and polarization parameters, while also correlating the electrochemical response with quantitative porosity, phase distribution, and elemental segregation.

6. The oxidation mechanism requires stronger experimental evidence because the lower mass gain of HPS is attributed to a denser microstructure and protective oxide layer; cross-sectional SEM/EDS, XRD or XPS analysis of the oxide scale, together with oxide-layer thickness and elemental distribution, should be provided to substantiate this proposed mechanism.

7. The authors may use the following article for the justification of ANN model in their study: Review of machine learning approaches for predicting mechanical behavior of composite materials

Reviewer #3: This study details the experimental, multiphysics, and artificial neural network-based analysis with geometry and cooling effects on an induction self-heating insert for rapid mold temperature control. Following revisions are required:

1. Avoid multiple paragraph breakage and multiple citations at a time. Please cite single sentence with a single reference only.

2. Most of the cited reference are outdated. Please provide the recent citation of 2024-2026.

3. Please report experimental uncertainty more clearly.

4. Provide stronger numerical model validation, the current form is not sufficient.

5. Clarify the ANN performance under flowing water.

6. Include a non-heated control for the molding test.

7. Discuss the ANN’s applicability beyond the tested geometries.

8. Compare the proposed cooling design with conventional channels.

**********

what does this mean?). If published, this will include your full peer review and any attached files.

If you choose “no”, your identity will remain anonymous but your review may still be made public.

**Do you want your identity to be public for this peer review?**  For information about this choice, including consent withdrawal, please see our Privacy Policy

Reviewer #1: No

Reviewer #2: No

Reviewer #3: No

**********

[NOTE: If reviewer comments were submitted as an attachment file, they will be attached to this email and accessible via the submission site. Please log into your account, locate the manuscript record, and check for the action link "View Attachments". If this link does not appear, there are no attachment files.]

To ensure your figures meet our technical requirements, please review our figure guidelines: https://journals.plos.org/plosone/s/figures

You may also use PLOS’s free figure tool, NAAS, to help you prepare publication quality figures: https://journals.plos.org/plosone/s/figures#loc-tools-for-figure-preparation.

NAAS will assess whether your figures meet our technical requirements by comparing each figure against our figure specifications.

# Author comment: Author response to Decision Letter 1

**DOI:** 10.1371/journal.pone.0359849.r002

31 Aug 2026

Response to Reviewer #1

Geometry and Cooling Effects on an Induction Self-Heating Insert for Rapid Mold Temperature Control: Experimental, Multiphysics, and Artificial Neural Network-Based Analysis

Dear Reviewer #1,

We thank you very much for the detailed technical comments. We revised the manuscript to report the archived COMSOL formulation and solver settings more transparently, clarify the cooling-boundary model, strengthen the description of ANN generalization, add available dimensional statistics for the molding demonstration, add the suggested recent literature, and reduce claims where the retained data do not support a controlled comparison. Where raw experimental or numerical data are no longer available, we state this explicitly rather than reconstructing unsupported values.

Comment 1

Reviewer comment: The electromagnetic–thermal model should provide the governing equations, mesh size, time-step sensitivity, convergence criteria, and temperature-dependent electrical/thermal properties used in the simulation.

Response: We agree that the numerical formulation required substantially more detail. The revised manuscript now identifies COMSOL Multiphysics 6.3, Magnetic Fields and Heat Transfer in Solids, and the sequential coupling in which the frequency-domain electromagnetic loss mf.Qh is transferred to the transient thermal model. The governing relations are given in Eqs. (1)–(5). We now report the retained free-tetrahedral mesh settings (maximum element size 11.6 mm, minimum element size 0.84 mm, maximum growth rate 1.4, curvature factor 0.4, and narrow-region resolution 0.7), the representative model degrees of freedom, and the retained adaptive BDF solver settings. The archived model used a constant soft-iron surrogate property set for the experimental C45 insert. Temperature-dependent electrical/thermal properties were not used. A formal mesh-independence study and a separate time-step sensitivity study were not retained with the archived model, so we do not claim that they were performed. This limitation is now stated explicitly.

Changes in the revised manuscript: Sections 2.4 and 4.3, Table 2, and the study-limitations paragraph were expanded. The manuscript now reports the actual retained model settings and explicitly identifies the absence of formal mesh/time-step sensitivity and temperature-dependent C45 properties.

Comment 2

Reviewer comment: The Taguchi L25 design should be presented explicitly, including the actual levels of T, W, and L, their physical meaning, and the rationale for selecting these ranges.

Response: We agree. The revised manuscript now states the physical meaning of T, W, and L and gives all five levels: T = 0.3, 0.5, 0.7, 0.9, and 1.1 mm; W = 30, 32.5, 35, 37.5, and 40 mm; and L = 76, 81, 86, 91, and 96 mm. We also clarify that these ranges correspond to the insert geometry set used in the study and vary the thin current-carrying/heating region while preserving the same outer insert envelope. The complete L25 matrix is provided in S1 Table.

Changes in the revised manuscript: Section 2.1.2 and Table 1 were revised; S1 Table contains the complete 25-case matrix.

Comment 3

Reviewer comment: The effects of cooling conditions (NW, SW, and FW) require quantitative analysis. Report water temperature, flow rate, Reynolds number, cooling-channel dimensions, and convective heat-transfer coefficients.

Response: We agree that the thermal boundary conditions needed to be described quantitatively. The archived COMSOL files show that the water was not modeled as a resolved CFD domain. NW was represented with the cooling-layer convective boundary disabled; SW used h = 200 W/(m²·K) at 298.15 K (25 °C); and FW used an effective forced-convection boundary with h = 2000 W/(m²·K) at 298.15 K. Because flow was not resolved, flow rate, Reynolds number, and Nusselt number are not model inputs and cannot be recovered from these files. We have revised the text so that FW is not presented as a CFD calculation.

Changes in the revised manuscript: Section 2.5 and Table 2 now distinguish prescribed convection from resolved water flow. The limitation is also discussed in Section 4.3.

Comment 4

Reviewer comment: Since the experimental ANN targets are based on the mean of five repeated measurements, the manuscript should report standard deviation/error bars and assess the repeatability and uncertainty of the temperature measurements.

Response: We agree in principle. The retained ANN dataset contains the mean temperature field from five repeats, but the five individual thermal fields are no longer available. Therefore, reconstructing pointwise SD or error bars would be statistically invalid. We now report the camera manufacturer-specified accuracy (approximately ±2 °C) separately from repeatability and explicitly state that this instrument accuracy is not a substitute for SD. We also state that repeat-level uncertainty cannot be reconstructed from the retained mean fields.

Changes in the revised manuscript: Sections 2.2, 2.8, and 4.5 were revised to distinguish instrument accuracy from repeatability and to state the limitation explicitly.

Comment 5

Reviewer comment: The reported ANN performance is relatively moderate, particularly R² = 0.493 for flowing water. The manuscript should avoid presenting the ANN as a highly predictive model and should discuss its limitations for design optimization.

Response: We agree. The revised manuscript explicitly describes the FW model as a moderate-prediction case. We retain the geometry-wise R² = 0.493 because this is the leakage-resistant result for unseen geometries. We also clarify that the full-25 model is used only for interpolation and screening within the investigated domain, not as evidence of independent accuracy or unrestricted optimization.

Changes in the revised manuscript: Sections 3.3, 4.4, 4.5, and the Conclusion were revised. The applicability domain is now stated explicitly.

Comment 6

Reviewer comment: These studies can be helpful for this work: (a) Computational analysis of polymer carbon nanotube composites using experiments and ANN modeling for manufacturing applications; (b) Investigation of tribological behaviour of B4C–graphene glass fiber composites using Taguchi, multi-criteria decision making, and machine learning approaches.

Response: Thank you for these suggestions. We reviewed both studies and added them to the revised manuscript because they provide recent examples of ANN modeling from experimental manufacturing data and Taguchi/ML sensitivity and interaction analysis.

Changes in the revised manuscript: The two suggested studies were added as Refs. 49 and 50 and cited in the ANN literature/discussion.

Comment 7

Reviewer comment: A sensitivity/ablation analysis should quantify the individual and interaction effects of T, W, L, X, Y, and cooling condition on predicted temperature.

Response: We agree that a formal feature-ablation and interaction analysis would provide additional interpretability. The locked analysis used three separate condition-specific ANN models and one-factor response sweeps after geometry-wise validation. Figs. 14–18 and Table 7 therefore quantify response trends for T, W, L, and cooling state, but they do not constitute a formal six-factor ablation/interactions decomposition. We did not create a post-hoc ablation result using a different model because it would not be directly comparable with the locked validation pipeline. The revised manuscript now states this distinction explicitly and identifies formal ablation analysis as future work.

Changes in the revised manuscript: Section 4.4 and the study-limitations paragraph were revised to state the scope of the existing factor analysis and the absence of formal feature ablation.

Comment 8

Reviewer comment: The proof-of-concept result of 28.6 µm spacing versus 30 µm nominal and 106.6 µm height versus 100 µm nominal should include measurement uncertainty, number of measured features, standard deviation, and measurement technique.

Response: We agree. The retained feature-level data allow the sample dispersion and number of measurements to be reported. Spacing was measured at four positions on each of two LDPE specimens (n = 8), giving 28.64 ± 1.98 µm (sample SD). Height was measured at five positions on each of two specimens (n = 10), giving 106.61 ± 4.43 µm (sample SD). The measurements were made with a Nikon NEXIV VMZ-R3020 non-contact CNC optical system; the retained equipment record gives a linear-scale resolution of 0.01 µm. We also clarify that these SD values describe dispersion among measured features/specimens and are not a complete traceable metrological uncertainty budget.

Changes in the revised manuscript: Sections 2.7 and 3.4, the Abstract, and the Conclusion were revised with n, sample SD, and measurement details.

Comment 9

Reviewer comment: The manuscript should quantify whether induction heating actually reduces cycle time, filling pressure, injection pressure, or frozen-layer thickness compared with conventional mold-temperature control.

Response: We agree that these quantities require a controlled comparison. The available molding demonstration does not include a non-heated control, and cycle time, injection/filling pressure, and frozen-layer thickness were not recorded as comparative outcomes. We therefore cannot quantify reductions in these variables without new experiments. Rather than infer unsupported improvements, we revised the manuscript to describe the molding test only as proof of feasibility and to remove quantitative comparative implications.

Changes in the revised manuscript: Sections 2.7, 3.4, 4.5, the Abstract, and the Conclusion now state explicitly that no cycle-time/pressure/frozen-layer reduction is claimed.

-------------------------------------------------------------------------------------------------------------------------------------------------------------------------------------------------------------------------------------

Response to Reviewer #2

Geometry and Cooling Effects on an Induction Self-Heating Insert for Rapid Mold Temperature Control: Experimental, Multiphysics, and Artificial Neural Network-Based Analysis

Dear Reviewer#2,

We appreciate the reviewer’s time. We respectfully note that Comments 1–6 appear to refer to a different manuscript. They discuss MA–HPS and VAM processing, BCC and Nb5Si3 phases, nanoscale grain size, corrosion, oxidation, hardness, and wear. None of these materials, processing routes, phases, or measurements are part of the submitted manuscript, which concerns an induction self-heating mold insert for injection molding. We have therefore not modified the manuscript in response to Comments 1–6 and have also informed the Editor. Comment 7 recommends a machine-learning review that is relevant to the ANN methodology, so we considered that reference separately.

Comment 1

Reviewer comment: The authors should provide a more rigorous quantitative analysis linking the processing routes (MA–HPS and VAM) with phase evolution, grain structure, porosity, and elemental segregation, rather than relying primarily on qualitative SEM observations.

Response: This comment appears to concern a different manuscript. The present study does not contain MA–HPS, VAM, SEM phase-evolution analysis, grain structure, porosity, or elemental segregation. No manuscript change was made for this item.

Changes in the revised manuscript: None; comment appears unrelated to the submitted manuscript.

Comment 2

Reviewer comment: The reported grain sizes of 25.1 nm for HPS and 46 nm for VAM require careful verification...

Response: This comment appears to concern a different manuscript. The submitted work does not report HPS/VAM grain sizes or XRD crystallite-size analysis.

Changes in the revised manuscript: None.

Comment 3

Reviewer comment: The phase identification of the BCC matrix and Nb5Si3 should be strengthened...

Response: This comment appears to concern a different manuscript. The submitted work contains no BCC/Nb5Si3 phase analysis or phase-fraction discussion.

Changes in the revised manuscript: None.

Comment 4

Reviewer comment: The mechanical-property comparison should include repeated measurements, standard deviations or error bars...

Response: This comment refers to yield strength, ultimate compressive strength, fracture strain, hardness, and wear rate, none of which are outcomes in the submitted manuscript. We have not made changes in response to these unrelated mechanical-property requests.

Changes in the revised manuscript: None.

Comment 5

Reviewer comment: The corrosion analysis should provide sufficient electrochemical testing details...

Response: The submitted manuscript contains no corrosion or electrochemical testing. This comment therefore appears to refer to a different study.

Changes in the revised manuscript: None.

Comment 6

Reviewer comment: The oxidation mechanism requires stronger experimental evidence...

Response: The submitted manuscript contains no oxidation testing, oxide-scale analysis, XPS, or oxidation mass-gain data. This comment therefore appears to refer to a different study.

Changes in the revised manuscript: None.

Comment 7

Reviewer comment: The authors may use the following article for the justification of ANN model in their study: Review of machine learning approaches for predicting mechanical behavior of composite materials.

Response: Thank you for this reference. Although the preceding comments appear unrelated to our manuscript, this review is relevant to general ANN methodology. We added it to support the discussion of data quality, overfitting, model interpretability, and validation limitations.

Changes in the revised manuscript: The suggested review was added as Ref. 51 and cited in the ANN discussion.

-------------------------------------------------------------------------------------------------------------------------------------------------------------------------------------------------------------------------------------

Response to Reviewer #3

Geometry and Cooling Effects on an Induction Self-Heating Insert for Rapid Mold Temperature Control: Experimental, Multiphysics, and Artificial Neural Network-Based Analysis

Dear Reviewer#3,

We thank you very much for the concise recommendations. The revision reduces grouped citations, emphasizes recent literature, clarifies experimental uncertainty and ANN limitations, expands the archived COMSOL formulation and solver details, and narrows the molding/cooling claims where a control experiment is not available.

Comment 1

Reviewer comment: Avoid multiple paragraph breakage and multiple citations at a time. Please cite single sentence with a single reference only.

Response: We revised the literature-heavy paragraphs so that individual claims are supported by a single reference per sentence rather than grouped citation blocks. We also consolidated related discussion to reduce unnecessary paragraph fragmentation.

Changes in the revised manuscript: The Introduction and Discussion literature paragraphs were rewritten throughout.

Comment 2

Reviewer comment: Most of the cited reference are outdated. Please provide the recent citation of 2024-2026.

Response: We retained a limited number of older references where they are foundational to induction heating and variotherm molding, but increased the use of recent literature. In addition to the existing 2024–2026 references, we added two 2026 studies suggested by Reviewer #1 and a 2025 machine-learning review.

Changes in the revised manuscript: Refs. 49–51 were added and the ANN literature discussion was updated.

Comment 3

Reviewer comment: Please report experimental uncertainty more clearly.

Response: We now distinguish the IR camera manufacturer-specified accuracy (approximately ±2 °C) from experimental repeatability. Because only the mean of five thermal repeats is retained, pointwise repeatability SD/error bars cannot be reconstructed. For the Chip 2 dimensional measurements, feature-level data are available and we now report n and sample SD.

Changes in the revised manuscript: Sections 2.2, 2.7, 2.8, 3.4, and 4.5 were revised.

Comment 4

Reviewer comment: Provide stronger numerical model validation, the current form is not sufficient.

Response: We strengthened the numerical section by reporting the actual archived multiphysics formulation, material properties, mesh settings, solver settings, cooling boundaries, and model assumptions. We also retain five representative experiment–COMSOL cases and additional comparisons in S2 and S3 Figs. However, full nodal fields are not available for all cases, so we do not introduce a global MAE/RMSE that cannot be reproduced. The model is now explicitly described as suitable for mechanism interpretation and design screening rather than metrological temperature prediction.

Changes in the revised manuscript: Sections 2.4, 3.2, 4.3, Table 2, Table 5, S2 Fig, S3 Fig, and S4 Table are referenced more explicitly.

Comment 5

Reviewer comment: Clarify the ANN performance under flowing water.

Response: We agree. FW is now explicitly described as a moderate-prediction case (R² = 0.493), not a highly predictive model. We explain that forced cooling flattens the temperature field and reduces the geometry-related signal relative to experimental/boundary variability.

Changes in the revised manuscript: Sections 3.3 and 4.4 were revised.

Comment 6

Reviewer comment: Include a non-heated control for the molding test.

Response: We agree that a non-heated control would be required for a causal comparison. Such a control was not included in the retained molding dataset and cannot be reconstructed retrospectively. We therefore revised the manuscript so that Fig. 19 is explicitly a proof-of-concept feasibility demonstration only and does not claim an improvement relative to non-heated molding.

Changes in the revised manuscript: Sections 2.7, 3.4, 4.5, the Abstract, and the Conclusion were revised.

Comment 7

Reviewer comment: Discuss the ANN’s applicability beyond the tested geometries.

Response: We now state the validated applicability domain explicitly: T = 0.3–1.1 mm, W = 30–40 mm, L = 76–96 mm, X = 0–130.5 mm, and Y = 0–88.5 mm under NW, SW, and FW. The full-25 models are described as interpolation/screening tools within this domain. Predictions outside these ranges are extrapolations and were not validated.

Changes in the revised manuscript: Section 4.4 was expanded.

Comment 8

Reviewer comment: Compare the proposed cooling design with conventional channels.

Response: We agree that a direct comparison with conventional straight channels would be useful. The current dataset does not include a matched conventional-channel benchmark. We therefore do not claim a quantified improvement in cooling time or uniformity over conventional channels. The discussion uses prior cooling-layer/conformal-cooling literature only as context, and the absence of a direct benchmark is now stated explicitly as a limitation.

Changes in the revised manuscript: Sections 3.3.4, 4.2, 4.5, and the Conclusion were revised

-------------------------------------------------------------------------------------------------------------------------------------------------------------------------------------------------------------------------------------

We appreciate your insightful comments, which have substantially improved the manuscript’s clarity and rigor. We attach the revised version (clean and tracked) together with this response letter.

With best regards,

On behalf of all co-authors

Faculty of Mechanical Engineering, HCM-UTE

Attachment Submitted filename: Response_to_Reviewer_3.docx

# Peer review: Decision Letter 1

**DOI:** 10.1371/journal.pone.0359849.r003

**Authors:** Gaurav Arora

21 Sep 2026

Geometry and Cooling Effects on an Induction Self-Heating Insert for Rapid Mold Temperature Control: Experimental, Multiphysics, and Artificial Neural Network-Based Analysis

PONE-D-26-40802R1

Dear Dr. Son Minh,

We’re pleased to inform you that your manuscript has been judged scientifically suitable for publication and will be formally accepted for publication once it meets all outstanding technical requirements.

Within one week, you’ll receive an e-mail detailing the required amendments. When these have been addressed, you’ll receive a formal acceptance letter and your manuscript will be scheduled for publication.

An invoice will be generated when your article is formally accepted. Please note, if your institution has a publishing partnership with PLOS and your article meets the relevant criteria, all or part of your publication costs will be covered. Please make sure your user information is up-to-date by logging into Editorial Manager at Editorial Manager® and clicking the ‘Update My Information' link at the top of the page. For questions related to billing, please contact billing support.

If your institution or institutions have a press office, please notify them about your upcoming paper to help maximize its impact. If they’ll be preparing press materials, please inform our press team as soon as possible -- no later than 48 hours after receiving the formal acceptance. Your manuscript will remain under strict press embargo until 2 pm Eastern Time on the date of publication. For more information, please contact onepress@plos.org.

Kind regards,

Gaurav Arora, Ph.D

Academic Editor

PLOS One

Additional Editor Comments (optional):

The reviewers have granted the acceptance.

Reviewers' comments:

Reviewer's Responses to Questions

**Comments to the Author**

Reviewer #1: All comments have been addressed

Reviewer #2: All comments have been addressed

Reviewer #3: All comments have been addressed

**********

2. Is the manuscript technically sound, and do the data support the conclusions?

Reviewer #1: Yes

Reviewer #2: Yes

Reviewer #3: Yes

**********

3. Has the statistical analysis been performed appropriately and rigorously?

Reviewer #1: Yes

Reviewer #2: Yes

Reviewer #3: Yes

**********

4. Have the authors made all data underlying the findings in their manuscript fully available?

The PLOS Data policy

Reviewer #1: Yes

Reviewer #2: Yes

Reviewer #3: Yes

**********

5. Is the manuscript presented in an intelligible fashion and written in standard English?

Reviewer #1: Yes

Reviewer #2: Yes

Reviewer #3: Yes

**********

Reviewer #1: All comments are addressed well.

Reviewer #2: Thank you for addressing all the comments I asked in the revision. You have done a good work. Keep researching.

Reviewer #3: The revised version of the research paper is addressed with all the suggestions. The revised version is acceptable in present form.

**********

what does this mean?). If published, this will include your full peer review and any attached files.

If you choose “no”, your identity will remain anonymous but your review may still be made public.

**Do you want your identity to be public for this peer review?**  For information about this choice, including consent withdrawal, please see our Privacy Policy

Reviewer #1: No

Reviewer #2: No

Reviewer #3: No

**********

# Editor report: Acceptance letter

**DOI:** 10.1371/journal.pone.0359849.r004

**Authors:** Gaurav Arora

PONE-D-26-40802R1

PLOS One

Dear Dr. Son Minh,

I'm pleased to inform you that your manuscript has been deemed suitable for publication in PLOS One. Congratulations! Your manuscript is now being handed over to our production team.

At this stage, our production department will prepare your paper for publication. This includes ensuring the following:

* All references, tables, and figures are properly cited

* All relevant supporting information is included in the manuscript submission,

* There are no issues that prevent the paper from being properly typeset

You will receive further instructions from the production team, including instructions on how to review your proof when it is ready. Please keep in mind that we are working through a large volume of accepted articles, so please give us a few days to review your paper and let you know the next and final steps.

Lastly, if your institution or institutions have a press office, please let them know about your upcoming paper now to help maximize its impact. If they'll be preparing press materials, please inform our press team within the next 48 hours. Your manuscript will remain under strict press embargo until 2 pm Eastern Time on the date of publication. For more information, please contact onepress@plos.org.

You will receive an invoice from PLOS for your publication fee after your manuscript has reached the completed accept phase. If you receive an email requesting payment before acceptance or for any other service, this may be a phishing scheme. Learn how to identify phishing emails and protect your accounts at https://explore.plos.org/phishing.

If we can help with anything else, please email us at customercare@plos.org.

Thank you for submitting your work to PLOS One and supporting open access.

Kind regards,

PLOS One Editorial Office Staff

on behalf of

Dr. Gaurav Arora

Academic Editor

PLOS One
